# Circulating cardiometabolic metabolite profiles associated with ambient air pollution and atrial fibrillation risk: a prospective cohort study

**DOI:** 10.1186/s12933-026-03129-9

**Published:** 2026-03-21

**Authors:** Tonghuan Shi, Chaojun Yang, Zhixing Fan, Marcel Sieme, Melina Tangos, Xingyue Wu, Miao Lin, Dizhe Huang, Benjamin Sasko, Jan Wintrich, Muchtiar Khan, Xinyi Liu, Assem Aweimer, Andreas Mügge, Ibrahim Akin, Loek van Heerebeek, Ibrahim El-Battrawy, Giuseppe Danilo Norata, Ulrich Schotten, Francesco Paneni, Kai Huang, Jian Yang, Nazha Hamdani

**Affiliations:** 1 Ruhr-University Bochum, Medical Faculty, Institute of Physiology, Department of Cellular and Translational Physiology, Bochum, Germany; 2 Department of Cardiology, The First College of Clinical Medical Sciences, China Three Gorges University, 443003 Yichang, China; 3 Hubei Key Laboratory of Ischemic Cardiovascular Disease, Yichang, 443003 China; 4 Hubei Provincial Clinical Research Center for Ischemic Cardiovascular Disease, Yichang, 443003 China; 5 Key Laboratory of Medical Electrophysiology of Ministry of Education, Medical Electrophysiological Key Laboratory of Sichuan Province, Institute of Cardiovascular Research, Southwest Medical University, Luzhou, 646000 China; 6 Department of Cardiology, Renmin Hospital of Wuhan University, Wuhan, 430060 China; 7 Medical Department II, Marien Hospital Herne, Ruhr University Bochum, Bochum, Germany; 8 Department of Cardiology, OLVG, Amsterdam, The Netherlands; 9 Department of Cardiology and Angiology, University Hospital Bergmannsheil, Ruhr University Bochum, Bochum, Germany; 10 Department of Cardiology and Angiology, University Hospital St. Josef- Hospital, Ruhr University Bochum, Bochum, Germany; 11 First Department of Medicine, University Medical Centre Mannheim (UMM), Mannheim, Germany; 12 Department of Pharmacological and Biomolecular Sciences, University of Milan, Via Balzaretti 9, Milan, Italy; 13 Center for the Study of Atherosclerosis, E. Bassini Hospital, Via Massimo Gorki 50, Cinisello Balsamo, Italy; 14 Department of Physiology, Cardiovascular Research Institute Maastricht, Maastricht University, Maastricht, The Netherlands; 15 Center for Translational and Experimental Cardiology (CTEC), Zurich University Hospital, University of Zurich, Zurich, Switzerland; 16 University Heart Center, University Hospital Zurich, University of Zurich, Zurich, Switzerland; 17 Hubei Key Laboratory of Metabolic Abnormalities and Vascular Aging, Huazhong University of Science and Technology, Wuhan, China; 18 Hubei Clinical Research Center of Metabolic and Cardiovascular Disease, Huazhong University of Science and Technology, Wuhan, China; 19 Clinical Center for Human Genomic Research, Union Hospital, Huazhong University of Science and Technology, Wuhan, China; 20 Department of Cardiometabolic Medicine, Renmin Hospital of Wuhan University, Wuhan, China; 21 State Key Laboratory of Metabolism and Regulation in Complex Organisms, College of Life Sciences, Wuhan University, Wuhan, China

**Keywords:** Air pollution, Atrial fibrillation, Metabolomic, Lipoprotein

## Abstract

**Background:**

Ambient air pollution has been linked to atrial fibrillation (AF), yet the underlying metabolic mechanisms remain poorly understood.

**Methods:**

We analyzed 227,324 UK Biobank participants without baseline AF. We constructed an air pollution score by aggregating all four pollutants (PM_2.5_, PM_10_, NO_2_, NO_x_). Nuclear magnetic resonance metabolomics identified a pollution-related metabolic signature through elastic net regression. Associations between air pollutants, the metabolic signature and AF were analyzed using Cox models. Mediation analysis was employed to examine the role of the metabolic signature in the association between air pollutants and AF.

**Results:**

During follow-up, 16,235 participants (7.14%) developed AF. We identified 65-metabolite signature significantly associated with air pollution, predominantly comprising lipoprotein lipid concentrations (32.31%), lipoprotein subclasses (15.38%), fatty acids (13.85%), and amino acids (12.31%). Each standard deviation increase in this metabolic signature was associated with 18% higher AF risk (HR = 1.18, 95%CI:1.03–1.35). The metabolic profile mediated 15.45% of the relationship between air pollution and AF, with lipoprotein parameters showing the strongest mediation effects.

**Conclusion:**

Air pollution-related metabolic signature was independently associated with AF risk and mediated a significant portion of pollution’s arrhythmogenic effects. These findings provide novel insights into biological mechanisms linking environmental exposures to AF.

**Graphical abstract:**

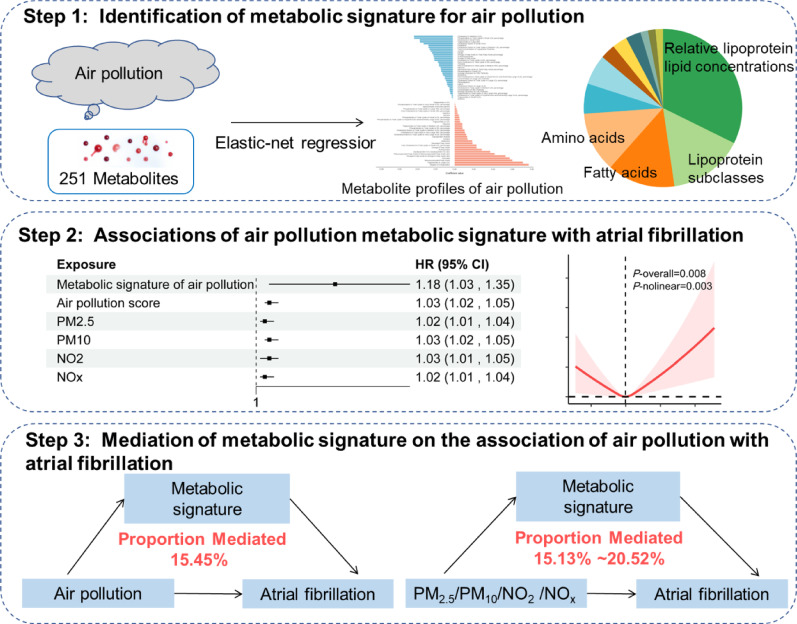

**Supplementary Information:**

The online version contains supplementary material available at 10.1186/s12933-026-03129-9.

## Research insights

**What is currently known about this topic?**

Ambient air pollution is associated with increased risk of atrial fibrillation (AF), and metabolic dysfunction is central to AF pathogenesis. Circulating metabolomic profiling has emerged as a useful tool to characterize biological responses to environmental exposures.

**What is the key research question?**

Is air pollution-related metabolomic signature associated with incident AF, and to what extent does it mediate the relationship between air pollution exposure and AF?

**What is new?**

A 65-metabolite signature associated with PM_2.5_, PM_10_, NO_2_, and NO_x_ was identified using elastic net regression in a large prospective cohort. This signature independently predicted AF risk and mediated approximately 15% of the association between air pollution and AF, with lipoprotein parameters showing the strongest mediation effects.

**How might this study influence clinical practice?**

Air pollution-related metabolic signature may improve AF risk stratification and identify potential metabolic targets for monitoring and prevention in populations exposed to air pollution.

## Introduction

Atrial fibrillation (AF) is the most common sustained cardiac arrhythmia and represents a significant public health burden worldwide. According to the Global Burden of Disease Study 2021, AF affected approximately 52.6 million people globally in 2021, with substantial increases in both incidence and mortality since 1990 [1]. The global incidence reached 4.48 million new cases in 2021, causing 0.34 million deaths and 8.36 million disability-adjusted life years [2]. Despite advancements in prevention and treatment strategies, the disease burden continues to grow, with projections indicating further increases through 2046 [3, 4]. Traditional risk factors for AF include high systolic blood pressure, which accounts for 34.0% of age-standardized AF deaths, followed by high body mass index (20.2%), alcohol use (7.4%), and smoking (4.3%) [2, 5]. Growing evidence suggests that environmental factors, particularly ambient air pollution, may play a significant role in AF pathogenesis beyond these established risk factors [6, 7].

Multiple studies have established significant associations between air pollution and AF. Notably, several large-scale investigations utilizing the UK Biobank database have provided evidence for this relationship. Ma et al. [8] found that long-term exposure to fine particulate matter 2.5 (PM_2.5_), particulate matter 10 (PM_10)_, nitrogen dioxide (NO_2_), and nitrogen oxides (NO_x_) significantly increased AF risk, with genetic susceptibility modifying these associations. Zhang et al. [9] further demonstrated that air pollution was associated not only with AF incidence but also with disease progression and complications. Additionally, Yang et al. [10] revealed that high PM_2.5_ exposure was associated with an 8% increased risk of AF, with effects modified by cardiovascular health status. Beyond the UK Biobank cohort, converging evidence from other populations supports these findings. A nationwide Korean study revealed that a 10 µg/m³ increase in PM_2.5_ was associated with 17.9% higher risk of new-onset AF [11]. Similarly, research from China involving over 1.3 million individuals found that PM_2.5_ and PM_10_ exposure was positively associated with AF prevalence [12]. Meta-analyses have confirmed these associations [13], though some studies report inconsistent findings [14]. Despite growing evidence supporting this relationship, the biological mechanisms underlying air pollution’s effects on AF remain incompletely understood.

Advances in nuclear magnetic resonance (NMR) metabolomics offer new insights into biological pathways linking air pollution and AF. NMR metabolomics enables objective evaluation of metabolic responses to environmental exposures [15, 16]. Recent studies have identified significant associations between circulating metabolites and specific air pollutants, with Hoffman et al. [17] developing a metabolomic risk score for traffic-related pollution and Xu et al. [18] identifying relationships with various lipid metabolites. Lifestyle-related metabolic profiles significantly impact cardiovascular outcomes [19]. While air pollution-related metabolomic signatures have been extensively studied in pulmonary [20], neurological [21], and hepatic diseases [22], whether these metabolic profiles can predict future AF risk remains largely unexplored, representing a critical knowledge gap in understanding the mechanisms linking environmental exposures to AF.

In this study, we sought to elucidate the metabolic mechanisms connecting ambient air pollution with AF using comprehensive metabolomic data from the UK Biobank. First, we constructed an air pollution score incorporating PM_2.5_, PM_10_, NO_2_, and NO_x_ to provide a holistic assessment of cumulative environmental exposure. We then identified and characterized a specific metabolic signature associated with this air pollution exposure. Subsequently, we prospectively examined whether this air pollution-related metabolic signature was independently associated with incident AF risk, and quantified its potential mediating effect in the air pollution-AF relationship.

## Methods

### Study design

The UK Biobank is a large-scale prospective cohort study designed to investigate genetic and environmental determinants of complex diseases. Between 2006 and 2010, approximately 502,132 participants aged 40–70 years were recruited from 22 assessment centers across England, Scotland, and Wales. The study collected comprehensive information on lifestyle factors, health status, physical measurements, and biological samples. Detailed information about the UK Biobank study protocol has been published previously and is available on the official website (www.ukbiobank.ac.uk). All participants provided written informed consent, and the study received ethical approval from the North West Multi-Centre Research Ethics Committee.

From the initial 502,132 UK Biobank participants, we excluded 227,895 individuals without measured NMR metabolomics data. Of the remaining 274,237 participants with metabolomics measurements, we further excluded 26,052 without completed NMR metabolomics markers and 17,272 without completed air pollution data. Additionally, we excluded 3589 participants with a history of AF at baseline. After applying these exclusion criteria, a total of 227,324 eligible participants were included in the final analysis (sFigure 1).

### Air pollution estimates

Annual average concentrations of ambient air pollutants, including PM_2.5_, PM_10_, NO_2_, and NO_x_, were estimated using Land Use Regression models developed through the European Study of Cohorts for Air Pollution Effects project [23, 24]. For PM_2.5_ and NOx, we utilized 2010 concentration data, while for PM_10_ and NO_2_, we calculated the average of available years (2007, 2010 for PM_10_; 2005–2007, 2010 for NO_2_) [25]. To comprehensively assess the cumulative exposure burden of multiple pollutants, we constructed an air pollution score by standardizing (z-score) and summing the concentrations of all four pollutants, with each pollutant weighted by its respective multivariable-adjusted risk estimate for AF obtained from preliminary analyses [20]. More detailed information on the air pollution estimation and air pollution score construction was summarized in the Supplemental Methods.

### NMR metabolomics measurements

Plasma metabolomic profiles measurements were performed by Nightingale Health platform (Helsinki, Finland) between June 2019 and April 2020 via high-throughput NMR spectroscopy on the stored non-fasting blood samples collected at baseline [26]. The standardized platform processed approximately 280,000 non-fasting EDTA plasma samples from UK Biobank participants, quantifying 251 metabolites including lipoprotein subclasses, fatty acids, amino acids, ketone bodies, and glycolysis-related metabolites. The platform provides absolute quantification in clinical units (mmol/L), enabling direct comparison with established biochemistry methods. Quality control procedures incorporated multiple internal standards to ensure measurement precision, with demonstrated high reproducibility across datasets. Detailed information on specific metabolites and their categorization is presented in sTable 1.

### Assessment of outcomes

Atrial fibrillation and flutter events, here merged as AF events, were identified through UK Biobank data linkages to national health registries with follow-up through December 2023. AF diagnoses were ascertained using multiple complementary data sources, including hospital admission records, death registries, and self-reported medical conditions. Specifically, we identified AF cases using ICD-9, ICD-10, procedural codes, and self-reported diagnoses. For each participant, follow-up time was calculated from the date of recruitment until the first occurrence of AF diagnosis, death, loss to follow-up, or the end of the study period, whichever came first. The detailed diagnostic codes used for AF identification are provided in sTable 2.

### Covariates measurement

Comprehensive baseline covariates were collected during the initial UK Biobank assessment (2006–2010) through standardized touchscreen questionnaires, verbal interviews, and physical measurements. Sociodemographic characteristics included age (years), sex (female or male), and race (categorized as White or Other). Anthropometric measurements were performed by trained personnel, with body mass index (BMI, kg/m^2^) calculated as weight divided by height squared.

Lifestyle factors were assessed through detailed questionnaires. Physical activity was categorized as low, moderate, or high based on the International Physical Activity Questionnaire. Smoking status was classified as never, previous, or current smoker. Alcohol consumption was similarly categorized as never, previous, or current. Dietary patterns were evaluated using the Dietary approaches to stop hypertension (DASH) score, which quantifies adherence to a heart-healthy eating pattern based on consumption of fruits, vegetables, whole grains, low-fat dairy, lean proteins, and reduced sodium and sugar intake.

Medical history was determined through a combination of self-reported information and linked health records. Participants reported physician-diagnosed conditions including diabetes mellitus, hypertension, and cancer. Cardiovascular disease (CVD) history (excluding AF) was defined as having a previous diagnosis of ischemic heart disease, stroke, or heart failure.

### Statistical analyses

Missing baseline covariates were imputed using multiple imputation by chained equations. Baseline characteristics were described by AF status, presenting continuous variables as mean ± standard (SD) deviation and categorical variables as frequencies (percentages). Both metabolic signatures and air pollution scores were standardized (z-scores) before further analysis. We constructed an air pollution-related metabolic signature employing elastic net regression, a machine learning approach that combines LASSO and ridge regression penalties. This method efficiently handles high-dimensional data with multicollinearity while promoting sparse models through feature selection. We regressed standardized metabolite concentrations against pollution levels, with optimal regularization parameters (α = 0.4, λ = 1 standard error) determined via 10-fold cross-validation minimizing mean squared prediction error. The final metabolic signature score was calculated as the weighted sum of selected metabolites with non-zero coefficients. Spearman correlation analysis assessed relationships between individual metabolites, metabolic signature, and air pollutants. The pollutants exposure and metabolic signature were also stratified by quintiles: low (the lowest quantile), intermediate (quantile 2–4), and high (the highest quantile) exposure.

Associations between air pollution, metabolic signature, and AF risk were evaluated using Cox proportional hazards models. We verified the proportional hazards assumption through graphical assessment of Schoenfeld residuals. Three progressive adjustment models were implemented: Model 1 adjusted for age, sex, and race; Model 2 further adjusted for BMI, physical activity, smoking status, alcohol consumption, and DASH score; Model 3 additionally adjusted for diabetes mellitus, hypertension, CVD, and cancer history. Exposure-response relationships and potential nonlinearities were examined using restricted cubic spline (RCS) analyses with knots at the 10th, 50th, and 90th percentiles.

Mediation analyses quantified the extent to which metabolic signature mediated the associations between air pollution and AF, employing the counterfactual framework implemented in the “CMAverse” package. Total effects were decomposed into direct and indirect effects. All mediation models adjusted for the full set of covariates from Model 3, satisfying assumptions regarding unmeasured confounding between exposure-outcome, mediator-outcome, and exposure-mediator relationships.

We performed subgroup analyses based on age, sex, race, and socioeconomic status, and assessed interaction analysis as statistical evidence of heterogeneity in the association across subgroup factors. Sensitivity analyses included: (1) complete-case analysis excluding participants with missing covariates; (2) excluding AF cases diagnosed within the first two years of follow-up to address potential reverse causation; (3) restricting analysis to participants without baseline chronic conditions (diabetes, hypertension, CVD, and cancer); (4) excluding participants who were taking a statin at baseline; and (5) using the 2010 air pollution concentrations; (6) further adjusting for traffic noise, availability of green space, and aspirin use; (7) using principal component analysis (PCA) to construct the air pollution score [20].

Statistical analyses were conducted using R version 4.4.2 (R Foundation for Statistical Computing, Vienna, Austria). Two-sided *P* < 0.05 was considered statistically significant, with false discovery rate correction applied to the analysis of individual plasma metabolites to control for multiple testing.

## Results

### Baseline characteristics of study participants

Table 1 presents the baseline characteristics of 227,324 UK Biobank participants stratified by incident AF status. During a median follow-up period of 13.35 years, 16,235 participants (7.14%) developed incident AF. Participants who developed AF were significantly older and predominantly male, had higher BMI and the history of diabetes mellitus, hypertension, CVD, and cancer, compared to those who remained AF-free. The mean air pollution score and pollutants was slightly higher in participants with incident AF compared to those without.

**Table 1:** Baseline characteristics of 227,324 UK biobank participants stratified by incident atrial fibrillation status

Characteristic	Overall (*n* = 227,324)	Participants without atrial fibrillation (*n* = 211,089)	Participants with atrial fibrillation (*n* = 16,235)
Age (years)	56.56 ± 8.08	56.15 ± 8.06	61.92 ± 6.05
*Sex (%)*			
Female	121,061 (53.25)	114,904 (54.43)	6157 (37.92)
Male	106,263 (46.75)	96,185 (45.57)	10,078 (62.08)
*Race (%)*			
White	215,991 (95.01)	200,153 (94.82)	15,838 (97.55)
Other	11,333 (4.99)	10,936 (5.18)	397 (2.45)
BMI (kg/m^2^)	27.54 ± 4.77	27.42 ± 4.70	29.08 ± 5.37
*Physical activity (%)*			
Low	43,424 (19.10)	40,107 (19.00)	3317 (20.43)
Moderate	91,154 (40.10)	84,878 (40.21)	6276 (38.66)
High	92,746 (40.80)	86,104 (40.79)	6642 (40.91)
*Smoke (%)*			
Never	124,583 (54.80)	117,280 (55.56)	7303 (44.98)
Previous	78,949 (34.73)	71,806 (34.02)	7143 (44.00)
Current	23,792 (10.47)	22,003 (10.42)	1789 (11.02)
*Alcohol (%)*			
Never	10,098 (4.44)	9390 (4.45)	708 (4.36)
Previous	8059 (3.55)	7354 (3.48)	705 (4.34)
Current	209,167 (92.01)	194,345 (92.07)	14,822 (91.30)
DASH	4.89 ± 1.34	4.89 ± 1.34	4.86 ± 1.36
*History of diabetes mellitus*			
No	215,044 (94.60)	200,580 (95.02)	14,464 (89.09)
Yes	12,280 (5.40)	10,509 (4.98)	1771 (10.91)
*History of hypertension (%)*			
No	157,521 (69.29)	150,006 (71.06)	7515 (46.29)
Yes	69,803 (30.71)	61,083 (28.94)	8720 (53.71)
*History of CVD (%)*			
No	211,966 (93.24)	198,647 (94.11)	13,319 (82.04)
Yes	15,358 (6.76)	12,442 (5.89)	2916 (17.96)
*History of cancer (%)*			
No	206,635 (90.90)	192,443 (91.17)	14,192 (87.42)
Yes	20,689 (9.10)	18,646 (8.83)	2043 (12.58)
Air pollution score	63.53 [58.68,68.60]	63.49 [58.68,68.60]	63.53 [58.76,68.57]
PM_2.5_ (µg/m^3^)	9.93 [9.29,10.57]	9.93 [9.29,10.57]	9.93 [9.29,10.58]
PM_10_ (µg/m^3^)	19.04 [17.97,20.24]	19.04 [17.96,20.24]	19.04 [17.99,20.20]
NO_2_ (µg/m^3^)	27.96 [23.02,33.62]	27.95 [23.01,33.63]	27.99 [23.06,33.47]
NO_x_ (µg/m^3^)	42.04 [34.12,50.66]	42.04 [34.11,50.65]	42.12 [34.30,50.78]

BMI, Body mass index; DASH, Dietary approaches to stop hypertension; CVD, Cardiovascular disease

### Identification of a metabolic signature for air pollution

Among air pollutants components (sTable 3), PM_2.5_ (HR = 1.02, 95% CI: 1.01–1.04), PM_10_ (HR = 1.03, 95% CI: 1.02–1.05), NO_2_ (HR = 1.03, 95% CI: 1.01–1.05), and NO_x_ (HR = 1.02, 95% CI: 1.01–1.04), showed significant associations with AF risk after full adjustment. The RCS model analysis of the associations of air pollution components with AF were showed in sFigure 2. Then, the air pollution score was generated as a summative weighted score based on the associations between different air pollution values and the risk of AF.

**Fig. 1:**
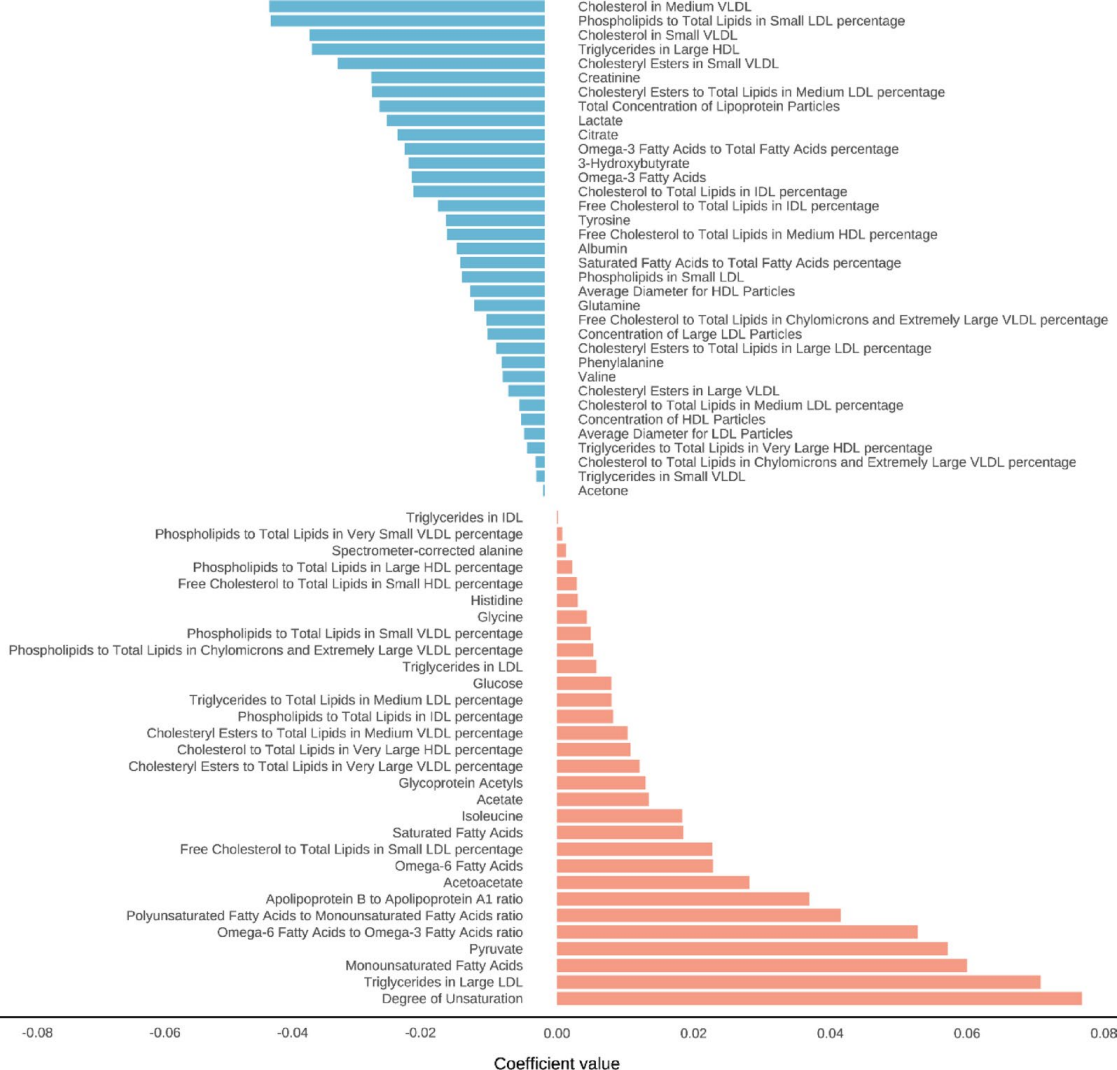
Metabolites ranked from the highest to the lowest elastic net positive and negative regression coefficients for air pollutant

Using elastic net regression, we identified 65 metabolites significantly associated with the air pollution score constructed from PM_2.5_, PM_10_, NO_2_, and NO_x_ (Fig. 1, sFigure 3). These selected metabolites represented diverse metabolic pathways, with the majority being relative lipoprotein lipid concentrations (32.31%, *n* = 21), followed by lipoprotein subclasses (15.38%, *n* = 10), fatty acids (13.85%, *n* = 9), and amino acids (12.31%, *n* = 8) (sFigure 4). Other identified metabolites included glycolysis-related metabolites (6.15%, *n* = 4), ketone bodies (6.15%, *n* = 4), fluid balance markers (3.08%, *n* = 2), lipoprotein particle concentrations (3.08%, *n* = 2), lipoprotein particle sizes (3.08%, *n* = 2), apolipoproteins (1.54%, *n* = 1), inflammation markers (1.54%, *n* = 1), and triglycerides (1.54%, *n* = 1).

**Fig. 2:**
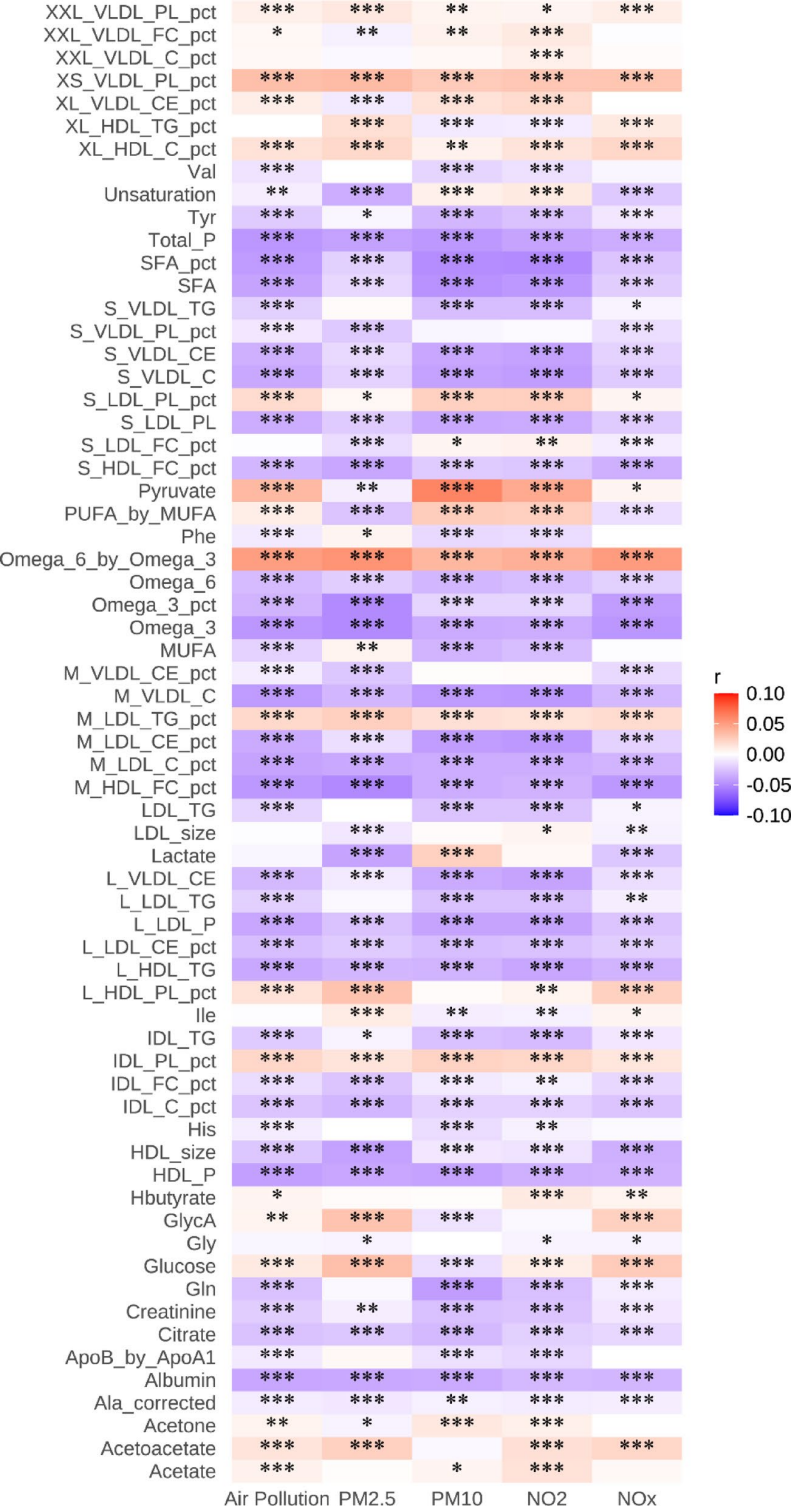
Correlation of the metabolic signature consisting of 65 selected metabolites with air pollution score and air pollution components ***P < 0.001, **P < 0.01, *P < 0.05

As shown in Fig. 1 and sTable 4, the metabolites with the strongest positive associations with air pollution included unsaturation, triglycerides in large low density lipoprotein (L_LDL_TG), monounsaturated fatty acids (MUFA), pyruvate, and omega-6 fatty acids to omega-3 fatty acids ratio (Omega_6_by_Omega_3). Conversely, the metabolites most negatively associated with air pollution were cholesterol in medium very low-density lipoprotein (M_VLDL_C), cholesterol in small very low-density lipoprotein (S_VLDL_C), Phospholipids to Total Lipids in small low-density lipoprotein percentage (S_LDL_PL_pct), Triglycerides in Large high density lipoprotein (L_HDL_TG), and cholesteryl esters in small very low-density lipoprotein (S_VLDL_CE). The correlation analysis demonstrated that most of these 65 metabolites had significant associations with the air pollution score and its individual components (PM_2.5_, PM_10_, NO_2_, and NO_x_), with varying degrees of statistical significance (Fig. 2). The levels of 65 metabolites in AF and non-AF groups were shown in sTable 5.

### Associations of air pollution and the related metabolic signature with atrial fibrillation

**Fig. 3:**
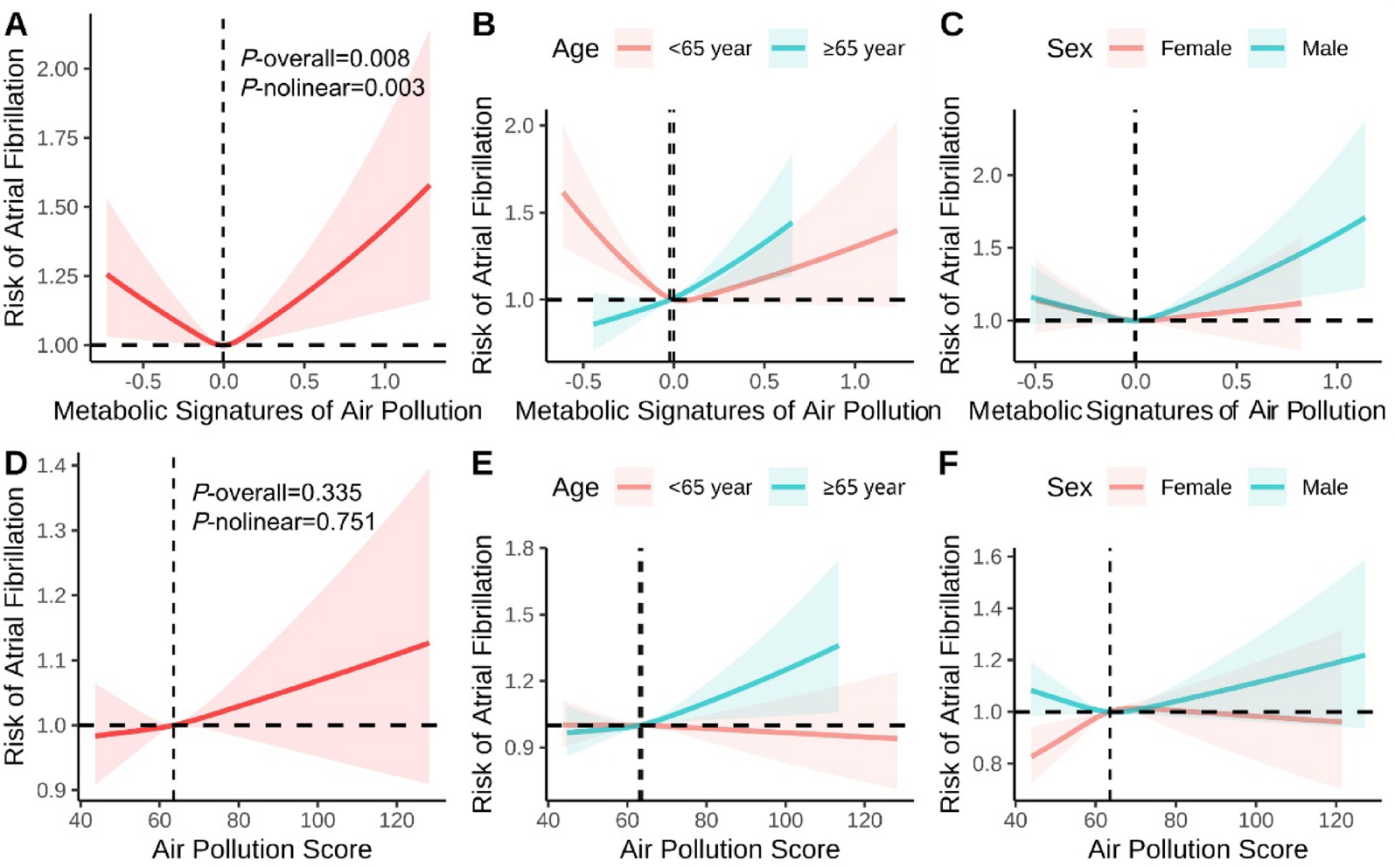
RCS analysis of the associations of air pollution score and the related metabolic profiles with atrial fibrillation Models were adjusted for age, sex, race, BMI, physical activity, smoke, alcohol, DASH, history of diabetes mellitus, hypertension, CVD, and cancer. BMI: Body mass index; DASH: Dietary approaches to stop hypertension; CVD: Cardiovascular disease

The associations of air pollution score, its components, and related metabolic signature with incident AF are presented in Table 2. After adjustment for age, sex, race, cardiovascular risk factors, and history of cancer (model 3), the air pollution score and its metabolic signature were significantly associated with increased AF risk. Each SD increment in the air pollution score was associated with a 3% higher risk of AF (HR = 1.03, 95% CI: 1.02–1.05), while each SD increment in the metabolic signature was associated with an 18% higher risk (HR = 1.18, 95% CI: 1.03–1.35). The receiver operating characteristic curve showed that the AUCs of air pollution metabolic characteristics in predicting the risk of AF occurrence at 5 years, 10 years, and 15 years were 0.771, 0.766 and 0.784, respectively (sFigure 5). When categorized into tertiles, participants with high exposure to air pollution had an 11% higher risk of AF compared to those with low exposure (HR = 1.11, 95% CI: 1.06–1.16), and those with high metabolic signature levels had a 14% higher risk (HR = 1.14, 95% CI: 1.05–1.19). The RCS analysis (Fig. 3) revealed non-linear relationships between the metabolic signature and AF risk (*P*-nonlinear = 0.003), while the relationship between the air pollution score and AF showed a more linear pattern (*P*-nonlinear = 0.751). The vertical dashed lines shown in the RCS plots represent the reference values used for spline modeling rather than statistically derived inflection points.

**Table 2:** Associations of air pollution score and the related metabolic profiles with atrial fibrillation

Exposure	Model 1		Model 2		Model 3	
	HR (95%CI)	*P*	HR (95%CI)	*P*	HR (95%CI)	*P*
*Metabolite profiles of air pollutants*						
Each SD increment	2.41 (2.12,2.74)	< 0.001	1.61 (1.41,1.83)	< 0.001	1.18 (1.03,1.35)	0.020
Low	Ref		Ref		Ref	
Medium	1.15 (1.11,1.19)	< 0.001	1.08 (1.04,1.12)	< 0.001	1.03 (0.99,1.06)	0.120
High	1.31 (1.26,1.37)	< 0.001	1.16 (1.11,1.21)	< 0.001	1.14 (1.05,1.19)	< 0.001
*P* for trend		< 0.001		< 0.001		0.010
*Air pollution score*						
Each SD increment	1.06 (1.04,1.08)	< 0.001	1.04 (1.02,1.06)	< 0.001	1.03 (1.02,1.05)	< 0.001
Low	Ref		Ref		Ref	
Medium	1.09 (1.05,1.13)	< 0.001	1.05 (1.01,1.08)	0.01	1.04 (1.00,1.07)	0.051
High	1.18 (1.13,1.24)	< 0.001	1.13 (1.08,1.18)	< 0.001	1.11 (1.06,1.16)	< 0.001
*P* for trend		< 0.001		< 0.001		< 0.001

Model 1 was adjusted for age, sex, and race;

Model 2 was adjusted for Model 1 + BMI, physical activity, smoke, alcohol, and DASH;

Model 3 was adjusted for Model 2 + history of diabetes mellitus, hypertension, CVD, and cancer

BMI, Body mass index; DASH, Dietary approaches to stop hypertension; CVD, Cardiovascular disease

The subgroup analyses (sTable 6) indicated that both the metabolic signature and air pollution score showed stronger associations with AF risk in males (HR = 1.43, 95% CI: 1.14–1.81; HR = 1.05, 95% CI: 1.02–1.08, respectively) than in females (HR = 1.08, 95% CI: 0.91–1.28; HR = 1.02, 95% CI: 1.00-1.04, respectively) with heterogeneity (*P*-interaction = 0.006). Similarly, participants aged ≥ 65 years showed stronger associations between both exposures and AF risk than those aged < 65 years (*P*-interaction < 0.001). Furthermore, the RCS analysis showed that the associations of air pollution and the metabolic signature with AF risk appeared stronger in males than in females and in older than in younger participants (Fig. 3).

Assessment of individual metabolites related to air pollution showed significant associations with AF risk for most metabolites (sTable 7). Among these, acetone (HR = 1.06, 95% CI: 1.05–1.07, *P* < 0.001), Free Cholesterol to Total Lipids in chylomicrons and extremely large VLDL percentage (XXL_VLDL_FC_pct) (HR = 1.06, 95% CI: 1.04–1.08), and polyunsaturated fatty acids (PUFA)_by_MUFA (HR = 1.07, 95% CI: 1.05–1.08) demonstrated the strongest positive associations. Conversely, several metabolites, particularly lipoprotein-related markers including cholesteryl esters in large VLDL (L_VLDL_CE) (HR = 0.89, 95% CI: 0.87–0.90), triglycerides in small VLDL (S_VLDL_TG) (HR = 0.90, 95% CI: 0.88–0.91), and S_VLDL_C (HR = 0.91, 95% CI: 0.90–0.93), showed significant protective associations against AF risk.

### Mediation of the metabolic signature on the association between air pollution and atrial fibrillation

**Fig. 4:**
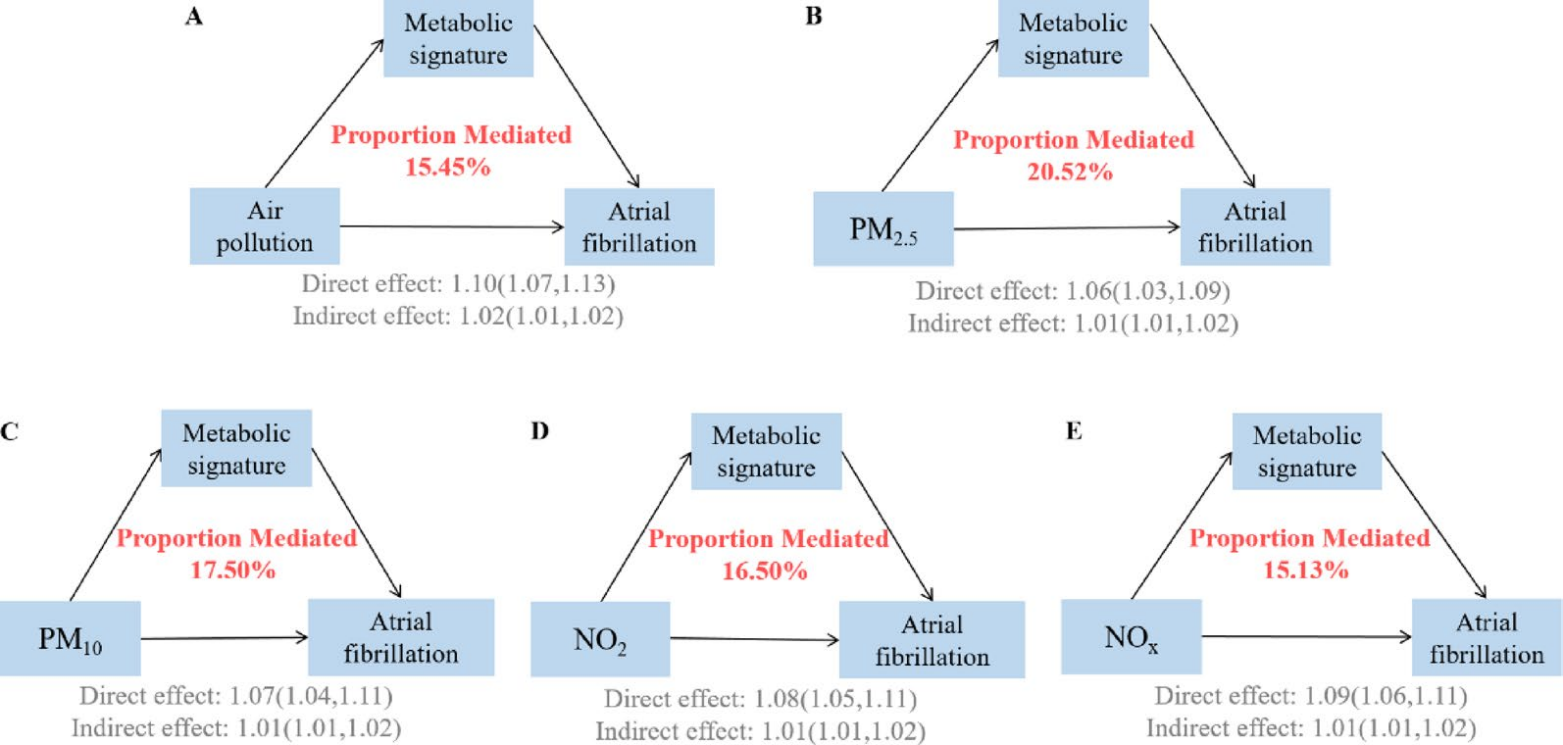
Mediation proportion of metabolic signature on the association of air pollution score and components with atrial fibrillation Models were adjusted for age, sex, race, BMI, physical activity, smoke, alcohol, DASH, history of diabetes mellitus, hypertension, CVD, and cancer. BMI: Body mass index; DASH: Dietary approaches to stop hypertension; CVD: Cardiovascular disease

As shown in Fig. 4, the identified metabolic signature significantly mediated the relationship between air pollution and AF incidence. The overall air pollution score’s effect on AF risk was mediated by the metabolic signature at 15.45% (95% CI: 11.84–20.77%) (sTable 8). Among individual pollutants, the metabolic signature mediated the largest proportion of the effect of PM_2.5_ (20.52%, 95% CI: 13.29–32.99%) on the risk of AF, followed by PM_10_ (17.50%, 95% CI: 12.21–26.89%), NO_2_ (16.50%, 95% CI: 12.79–23.73%), and NO_x_ (15.13%, 95% CI: 12.09–20.74%).

Further analysis revealed that among the 65 air-pollution-related metabolites, 53 significantly mediated the relationship between air pollution and AF (sTable 9). Lipoprotein-related parameters exhibited substantial mediation effects, with the strongest contributions from albumin (9.48%, 95% CI: 8.58–13.53%), phospholipids to total lipids in very small VLDL percentage (XS_VLDL_PL_pct) (9.21%, 95% CI: 7.87–11.83%), concentration of large LDL particles (L_LDL_P) (9.22%, 95% CI: 7.57–12.94%), M_VLDL_C (10.69%, 95% CI: 9.10–14.68%), and Free Cholesterol to Total Lipids in Medium HDL percentage (M_HDL_FC_pct) (9.37%, 95% CI: 8.49–12.22%). Other significant mediators included Omega-3 fatty acids (7.47%, 95% CI: 5.21–10.98%), omega-6 fatty acids (8.15%, 95% CI: 6.57–11.19%), and total phospholipids (7.40%, 95% CI: 6.30–9.89%).

### Sensitive analysis

To validate our findings, we performed five sensitivity analyses. First, when excluding participants with missing covariates (sTable 10, 11), the association between the metabolic signature and AF remained significant (per-SD HR: 1.12, 95% CI: 1.05–1.32; high vs. low: HR: 1.08, 95% CI: 1.02–1.11), with similar mediation effects (15.12%). Second, excluding incident AF cases within two years of follow-up (sTable 12, 13) yielded consistent results (per-SD HR: 1.22, 95% CI: 1.06–1.41; high vs. low: HR: 1.07, 95% CI: 1.02–1.12; mediation: 14.92%). Third, in participants without baseline chronic diseases (sTable 14, 15), although the mediating effect was slightly weakened, the association was slightly stronger (per-SD HR: 1.33, 95% CI: 1.05–1.68; mediation: 11.17%). Fourth, after excluding participants who were taking a statin at baseline (sTable 16, 17) yielded consistent results (per-SD HR: 1.27, 95% CI: 1.07–1.50; mediation: 11.01%). Fifth, when using the air pollution in 2010 (sTable 18, 19), the metabolic characteristics and the risk of AF did not change, but the mediating effect was enhanced (per-SD HR: 1.18, 95% CI: 1.02–1.36; mediation: 28.69%). Sixth, after further adjustment for traffic noise, availability of green space, and aspirin use, the associations and mediation effects remained robust (sTable 20, 21). Seventh, we used PCA to construct an alternative air pollution score, and the results were materially consistent (sTable 22, 23).

## Discussion

In this large-scale prospective UK Biobank cohort study, we identified a 65-metabolite signature associated with ambient air pollution exposure that independently predicted AF risk. We demonstrated for the first time that this metabolic signature significantly mediated the pollution-AF relationship, explaining approximately 15.45% of this association. Lipoprotein-related parameters showed the strongest mediation effects among all metabolites. These findings provide novel insights into biological mechanisms that may link environmental air pollution to AF risk and identify potential metabolic targets that could be useful for monitoring and preventing air pollution-related CVD.

Numerous previous studies have assessed metabolomic signatures associated with ambient air pollution exposure. Hoffman et al. [17] developed a metabolomic risk score for traffic-related air pollution exposure, further supporting the biological plausibility of metabolic responses to environmental exposures. Liang et al. [27] identified 95 metabolites linked to air pollutants, enriched in oxidative stress and inflammatory pathways. Compared with these two studies, our research has a larger sample size, and the elastic net regression method we used better handles multicollinearity among metabolites. Several studies have examined air pollution-related metabolic signatures in other disease contexts. Yang et al. [20] identified a 106-metabolite signature that mediated 10.5% of pollution-chronic obstructive pulmonary disease associations. Wang et al. [28] and Ran et al. [22] similarly identified metabolic signatures associated with PM_2.5_, PM_10_, NO_2_, and NO_x_ that were associated with disease risk and mediated 5.71–12.71% of the pollution-disease relationship. Although our study identified 65 metabolites—fewer than some previous investigations—the mediation proportion of 15.45% observed for AF exceeds those reported for other disease outcomes, suggesting that metabolic dysregulation may represent an important mechanistic pathway in air pollution-induced AF.

Previous research has extensively evaluated metabolic factors associated with AF and the metabolomic manifestations of this arrhythmia. Wijdeveld et al. [29] conducted a comprehensive review and meta-analysis of metabolomics in AF, identifying consistent alterations in fatty acid metabolism, energy substrate utilization, and inflammatory pathways. Zheng et al. [30] identified 123 NMR-measured analytes associated with CVD, particularly lipoprotein-related parameters similar to those identified in our study. Van Vugt et al. [31] identified 35 metabolites associated with cardiac disease, with AF particularly affected by phosphatidylcholines, amino acids, and acylcarnitines—metabolite classes that featured prominently in our air pollution-related signature. Zhang et al. [32] utilized metabolomics to characterize AF, validating arachidonic acid, glycolic acid, and palmitelaidic acid as potential AF biomarkers. This aligns with our findings that polyunsaturated fatty acids were strongly associated with both air pollution exposure and AF risk. Mayr et al. [33] demonstrated that ketone body metabolism is altered in persistent AF, which corresponds with our identification of ketone bodies among the air pollution-related metabolites. Furthermore, our study extends prior research by bridging environmental exposure and AF metabolomics, demonstrating that air pollution-related metabolic signature, particularly lipoprotein parameters, not only associates with AF risk but also significantly mediate the pollution-AF relationship. These findings suggest that environmental pollutants may contribute to AF development through exacerbating intrinsic metabolic vulnerabilities.

Our research findings suggest a complex pathophysiological cascade that may link environmental air pollution to AF through specific metabolic alterations. Particulate matter exposure triggers arrhythmogenic conditions through three key pathways: oxidative stress, autonomic dysfunction, and direct myocardial mitochondrial damage [34]. Our metabolomic analysis indicates that air pollution influences AF primarily through alterations in lipid metabolism, fatty acid metabolism, amino acid metabolism, and energy metabolic pathways. Lipoprotein dysregulation, particularly changes in LDL and HDL parameters, directly affects atrial myocyte membrane composition and function, increasing susceptibility to AF [35]. Alterations in fatty acid metabolism and mitochondrial energetics directly promote the development of AF substrate through electrical and structural remodeling [36]. The balance of polyunsaturated fatty acids is crucial for atrial electrical stability, with omega-6 fatty acids exhibiting protective effects against AF, likely related to their anti-inflammatory and antioxidant properties [37]; while Omega-3 fatty acids may reduce AF occurrence by modulating calcium handling and membrane stability [38]. Mitochondria play a central role in AF pathogenesis, and air pollution-induced mitochondrial dysfunction leads to energy deficiency and abnormal calcium handling, explaining the alterations in Krebs cycle metabolites observed in our signature [39]. Additionally, the alterations in amino acid networks in AF that we observed influence cellular bioenergetics and membrane stability, contributing to AF development [40]. Atrial metabolic stress directly increases AF vulnerability through altered glucose utilization and lipotoxicity [41]. Our findings provide crucial molecular evidence of the air pollution-metabolism-AF connection, shedding light on potential pathways through which environmental factors may be associated with arrhythmogenesis.

The observation that the combined air pollution score showed a lower mediation proportion than the individual pollutants warrants careful consideration. This counterintuitive finding likely reflects the complex interplay of multiple pollutants through divergent metabolic pathways. Different air pollutants may trigger distinct biological mechanisms: PM components primarily affect oxidative stress and lipid metabolism pathways [42], while gaseous pollutants like NO_2_ and NO_x_ may predominantly influence inflammatory and amino acid metabolism pathways [43]. When aggregated into a combined score, these pollutants with partially distinct or overlapping metabolic effects may result in attenuated overall mediation effects. Multicollinearity among pollutants was moderate (VIF range: 2.6–3.9), indicating acceptable correlations without severe model instability. Our metabolic signature was constructed using elastic net regression, which effectively addresses multicollinearity among highly correlated pollutants by applying both L1 and L2 penalties to simultaneously perform variable selection and coefficient shrinkage. This regularization approach ensures that the signature captures the most robust metabolic features while minimizing bias from correlated exposures [44]. The lower mediation proportion of the combined score does not necessarily indicate reduced biological relevance, but rather reflects that aggregating exposures with heterogeneous pathway-specific effects may mask individual pollutant-specific contributions. Future studies employing pathway-specific mediation analysis could further elucidate these complex pollutant-metabolite-disease relationships.

Our findings have significant implications for public health, clinical practice, and basic research. From a public health perspective, the identification of specific metabolic mediators may strengthen the mechanistic evidence for more stringent air pollution control policies, particularly in areas with high CVD burden [45]. Clinically, this air pollution-related metabolic signature may serve as a panel of early biomarkers for AF risk stratification, and could potentially improve prevention strategies through targeted metabolic interventions in susceptible individuals [34]. The strong mediation effect observed with lipoprotein-related parameters suggests that lipid-modifying therapies might be especially beneficial for those with high pollution exposure. For basic research, our findings provide a framework for investigating the broader implications of environmental exposures on cardiovascular health through metabolic alterations, highlighting potential therapeutic targets at the intersection of environmental cardiology and metabolism.

Our study features several key strengths. We utilized comprehensive NMR metabolomics data from a large prospective cohort with extensive follow-up, enhancing the robustness of our findings. We developed a holistic air pollution score incorporating multiple pollutants (PM_2.5_, PM_10_, NO_2_, and NO_x_), providing more comprehensive environmental exposure assessment than single-pollutant approaches. Importantly, we employed elastic net regression, which efficiently handles high-dimensional metabolomics data with multicollinearity while promoting sparse model selection, enabling us to identify a specific 65-metabolite signature with strong predictive value for AF development (18% higher risk per SD increment). Our mediation analysis quantitatively demonstrates that metabolic alterations explain approximately 15% of the air pollution-AF association, providing novel mechanistic insights.

There are several limitations that warrant consideration. First, our air pollution exposure assessment was based on residential addresses and did not account for individual time–activity patterns (including time spent outdoors), workplace exposure, or indoor air quality, which may result in exposure misclassification. Variations in individual time–activity patterns could introduce measurement error in estimating actual personal exposure levels, potentially attenuating the observed associations between air pollution and health outcomes. In addition, air pollution levels are dynamic and may vary over time due to seasonal fluctuations, long-term trends, and policy-driven changes. The exposure estimates used in this study were derived from specific calendar years and may not fully capture temporal variations in long-term ambient pollution levels throughout the follow-up period. Although most current studies on the association between air pollution exposure and outcomes have difficulty taking into account residential mobility during follow-up, we recognize that such mobility may introduce exposure misclassification and affect exposure assessment. Second, the NMR metabolomics platform, while comprehensive, captures only a subset of the human metabolome, and important metabolites relevant to air pollution or AF might have been missed. It is critical to note that the higher mediation proportion observed for lipid-related molecules may partly reflect measurement bias of the NMR platform, which primarily targets lipids, rather than indicating their biological predominance in the air pollution-AF pathway or true biological importance. Third, there may be deficiencies in the diagnosis of AF. In our study, we mainly identified patients with new-onset AF through hospitalization and cause-of-death data. Community-diagnosed AF cases may have been missed, introducing potential bias [46]. Fourth, despite adjusting for multiple confounders, residual confounding from unmeasured factors such as medication use, detailed dietary patterns, or genetic susceptibility cannot be ruled out. In addition, other environmental exposures, such as ozone and meteorological factors (e.g., temperature and humidity), were not available in the current dataset and may also contribute to residual confounding. Fifth, our findings from the UK Biobank may not be fully generalizable to other populations with different environmental exposures, genetic backgrounds, or lifestyle factors. Furthermore, the UK Biobank cohort comprises predominantly white participants. The limited racial/ethnic diversity restricts the external validity of our findings and their applicability to more diverse populations. Sixth, the observational nature of our study precludes definitive causal inference; therefore, future studies employing Mendelian randomization and experimental designs are warranted to strengthen causal inference and to further elucidate the underlying biological mechanisms. Seventh, while we demonstrated significant mediation effects, approximately 85% of the association between air pollution and AF remains unexplained, suggesting other important biological pathways warrant exploration.

## Conclusion

In conclusion, using a large prospective cohort design and an elastic net regression model, we identified a 65-metabolite signature, predominantly characterized by alterations in lipoprotein metabolism, fatty acids, and amino acids, that was independently associated with increased AF risk. This metabolic signature explained approximately 15% of the association between air pollution exposure and AF incidence, with lipoprotein-related parameters showing the strongest mediation effects. Our findings highlight the importance of metabolic pathways as biological mechanisms linking environmental exposures to AF.

## Supplementary Information

Below is the link to the electronic supplementary material.

Supplementary Material 1

## Data Availability

The data are available from the UK Biobank, but there are restrictions on their availability. Researchers who wish to access the UK Biobank database will need to apply for access through the following link: [https://www.ukbiobank.ac.uk/enable-your-research/](https:/www.ukbiobank.ac.uk/enable-your-research) .

## Abbreviations

AF: Atrial fibrillation
BMI: Body mass index
CVD: Cardiovascular disease
DASH: Dietary approaches to stop hypertension
HDL: High-density lipoprotein
LDL: Low-density lipoprotein
MUFA: Monounsaturated fatty acids
NMR: Nuclear magnetic resonance
NO_2_: Nitrogen dioxide
NO_X_: Nitrogen oxides
PCA: Principal component analysis
PM_2.5_: Particulate matter 2.5
PM_10_: Particulate matter 10
PUFA: Polyunsaturated fatty acids
RCS: Restricted cubic spline
SD: Standard deviation
VLDL: Very low-density lipoprotein
