# Supplementary material for: Circulating cardiometabolic metabolite profiles associated with ambient air pollution and atrial fibrillation risk: a prospective cohort study
Source: Cardiovasc Diabetol. 2026 Mar 21;25:133. doi: 10.1186/s12933-026-03129-9 (PMC13130729; doi:10.1186/s12933-026-03129-9)
Supplement: Supplementary file 1 — Supplementary Material 1 [file 12933_2026_3129_MOESM1_ESM.docx]

**Circulating Cardiometabolic Metabolite Profiles Associated with Ambient Air Pollution and Atrial Fibrillation Risk: A Prospective Cohort Study**

Supplement

[Air pollution estimates 2](#_Toc221699511)

[sFigure 1. Flow chart for study population selection. 3](#_Toc221699512)

[sFigure 2. RCS analysis of the associations of air pollution components with atrial fibrillation 4](#_Toc221699513)

[sFigure 3. Screening of metabolic signature for air pollution by elastic network regression. 5](#_Toc221699514)

[sFigure 4. Classification of 65 metabolic signature of air pollution screened by elastic network regression. 5](#_Toc221699515)

[sFigure 5. ROC curve of air pollution related metabolic profiles predicting the risk of atrial fibrillation. 6](#_Toc221699516)

[sTable 1. Description and group of metabolite by NMR in UK Biobank. 6](#_Toc221699517)

[sTable 2. Definitions of atrial fibrillation and other disease. 15](#_Toc221699518)

[sTable 3. Associations of air pollution components with atrial fibrillation. 23](#_Toc221699519)

[sTable 4. Elastic net regression coefficients of the 65 metabolites included in the air pollution-related metabolic signature. 23](#_Toc221699520)

[sTable 5. Median and Interquartile range of concentrations for the 65 metabolites quantified by NMR. 25](#_Toc221699521)

[sTable 6. Subgroup of the associations of air pollution score and the related metabolic profiles with atrial fibrillation. 26](#_Toc221699522)

[sTable 7. Cox regression model to assess associations between 65 metabolites of air pollution and atrial fibrillation. 28](#_Toc221699523)

[sTable 8. Mediation of metabolic signature on the association of air pollution score and components with atrial fibrillation. 29](#_Toc221699524)

[sTable 9. Mediation proportion of 65 air pollution metabolites on the association of air pollution score with atrial fibrillation. 30](#_Toc221699525)

[sTable 10. Sensitive analysis of the associations of air pollution score and the related metabolic profiles with atrial fibrillation after exclusion of participants with missing covariates. 32](#_Toc221699526)

[sTable 11. Sensitive analysis of the mediation of metabolic signature on the association of air pollution score and components with atrial fibrillation after exclusion of participants with missing covariates. 32](#_Toc221699527)

[sTable 12. Sensitive analysis of the associations of air pollution score and the related metabolic profiles with atrial fibrillation after exclusion of participants who developed atrial fibrillation within 2 years of follow-up. 33](#_Toc221699528)

[sTable 13. Sensitive analysis of the mediation of metabolic signature on the association of air pollution score and components with atrial fibrillation after exclusion of participants who developed atrial fibrillation within 2 years of follow-up. 34](#_Toc221699529)

[sTable 14. Sensitive analysis of the associations of air pollution score and the related metabolic profiles with atrial fibrillation after exclusion of participants who has the history of chronic disease. 34](#_Toc221699530)

[sTable 15. Sensitive analysis of the mediation of metabolic signature on the association of air pollution score and components with atrial fibrillation after exclusion of participants who has the history of chronic disease. 35](#_Toc221699531)

[sTable 16. Sensitive analysis of the associations of air pollution score and the related metabolic profiles with atrial fibrillation after excluding participants who were taking a statin at baseline. 36](#_Toc221699532)

[sTable 17. Sensitive analysis of the mediation of metabolic signature on the association of air pollution score and components with atrial fibrillation after excluding participants who were taking a statin at baseline. 36](#_Toc221699533)

[sTable 18. Sensitive analysis of the associations of air pollution score and the related metabolic profiles with atrial fibrillation after using the 2010 air pollution concentrations. 37](#_Toc221699534)

[sTable 19. Sensitive analysis of the mediation of metabolic signature on the association of air pollution score and components with atrial fibrillation after using the 2010 air pollution concentrations. 37](#_Toc221699535)

[sTable 20. Sensitive analysis of the associations of air pollution score and the related metabolic profiles with atrial fibrillation after further adjusting for traffic noise, availability of green space, and aspirin use. 38](#_Toc221699536)

[sTable 21. Sensitive analysis of the mediation of metabolic signature on the association of air pollution score and components with atrial fibrillation after further adjusting for traffic noise, availability of green space, and aspirin use. 39](#_Toc221699537)

[sTable 22. Sensitive analysis of the associations of air pollution score and the related metabolic profiles with atrial fibrillation after using PCA to construct the air pollution score.. 40](#_Toc221699538)

[sTable 23. Sensitive analysis of the mediation of metabolic signature on the association of air pollution score and components with atrial fibrillation after using PCA to construct the air pollution score. 40](#_Toc221699539)

Air pollution estimates

The air pollution exposure data utilized in this study were acquired from the Small Area Health Statistics Unit as part of the BioSHaRE-EU Environmental Determinants of Health Project and the UK Department for Environment, Food and Rural Affairs (DEFRA). We assessed ambient air pollution concentrations using advanced Land Use Regression (LUR) models developed within the framework of the European Study of Cohorts for Air Pollution Effects (ESCAPE) project [1, 2]. These sophisticated models integrated various geographic information system-derived variables including traffic intensity, road network density, land use classification, population distribution, and topographic characteristics to capture fine-scale spatial variations in pollutant levels. Individual exposure assessment was conducted by geocoding participants' residential addresses and intersecting these coordinates with high-resolution pollution concentration grids (100m × 100m). For participants reporting residential relocations during the study period, we calculated time-weighted average exposures based on duration at each address. Our analysis incorporated data on PM2.5 (available for 2010), PM10 (available for 2007 and 2010), NO2 (available for 2005-2007 and 2010), and NOx (available for 2010) [3]. Following methodological conventions, we utilized averaged concentrations across available years for PM10 and NO2, while employing 2010 data for PM2.5 and NOx. Model validation through rigorous leave-one-out cross-validation procedures demonstrated robust predictive performance with R² values of 77% for PM2.5, 60% for PM10, 85% for NO2, and 67% for NOx.

To holistically capture the combined impact of multiple pollutants, we developed a comprehensive air pollution score by standardizing and aggregating all four pollutants (PM2.5, PM10, NO2, and NOx), with each component weighted according to its respective strength of association with atrial fibrillation in preliminary multivariable-adjusted analyses [4].


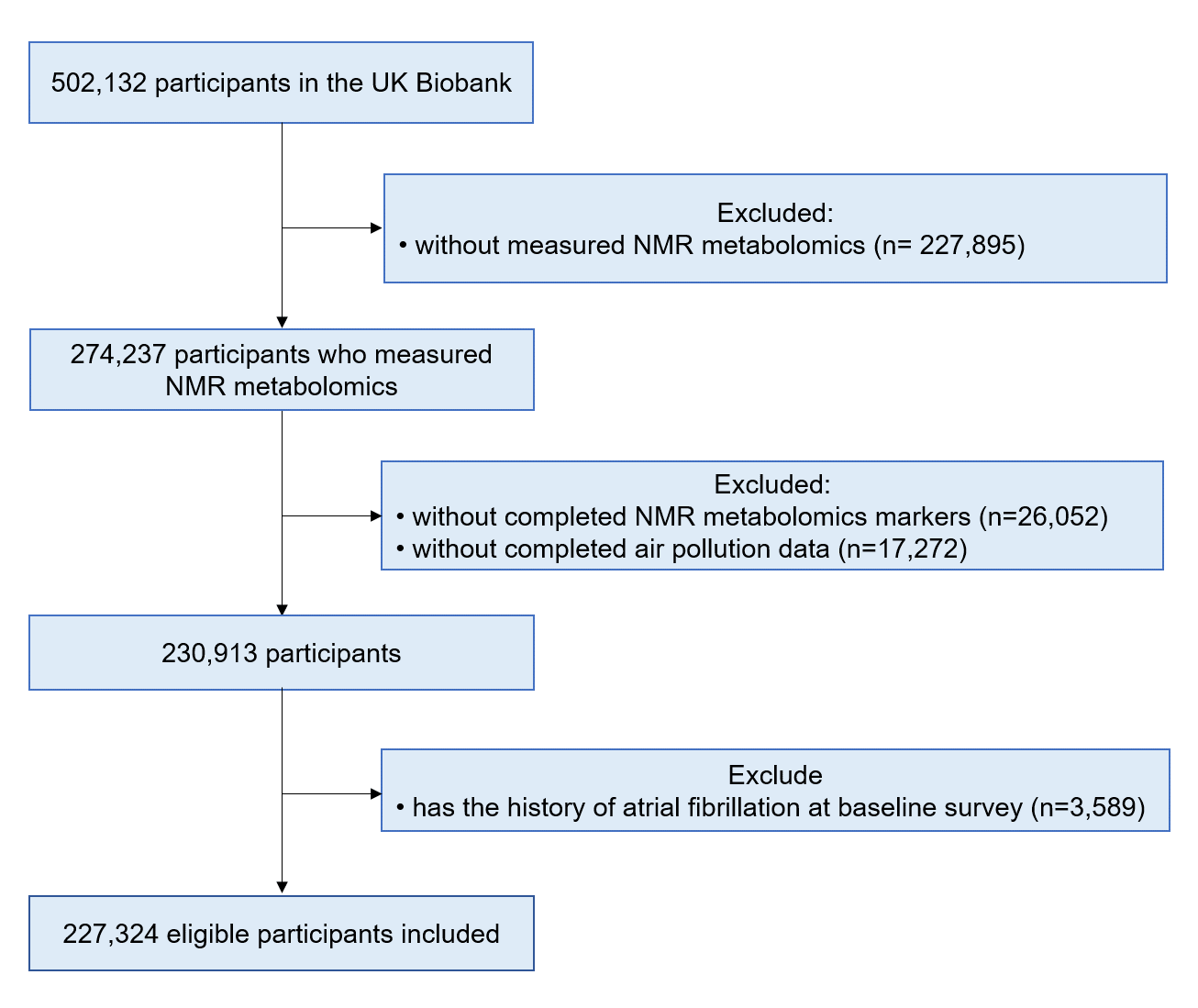


sFigure 1. Flow chart for study population selection.


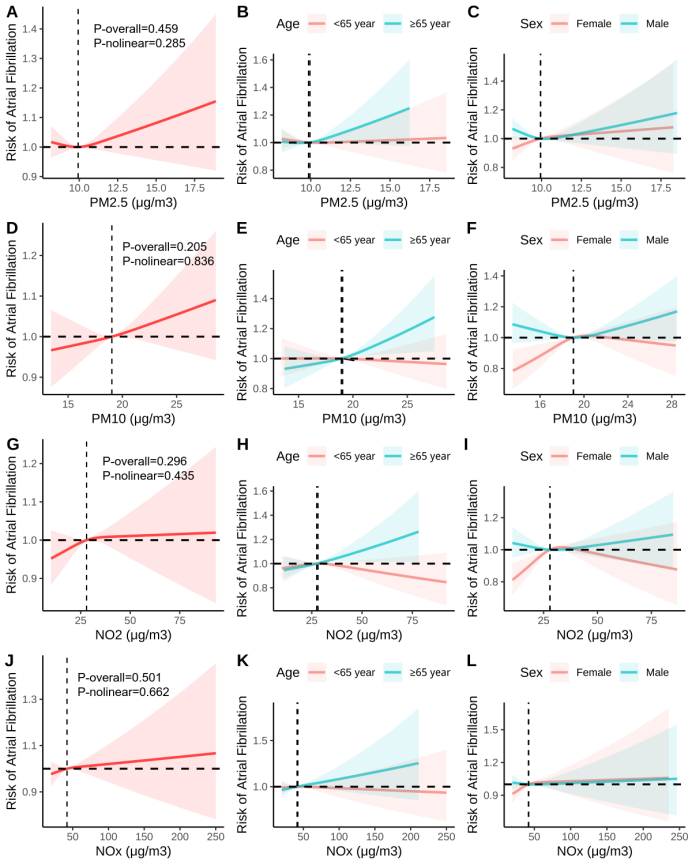


sFigure 2. RCS analysis of the associations of air pollution components with atrial fibrillation

Models were adjusted for age, sex, race, BMI, physical activity, smoke, alcohol, DASH, history of diabetes mellitus, hypertension, CVD, and cancer. BMI: Body mass index; DASH: Dietary approaches to stop hypertension; CVD: Cardiovascular disease.


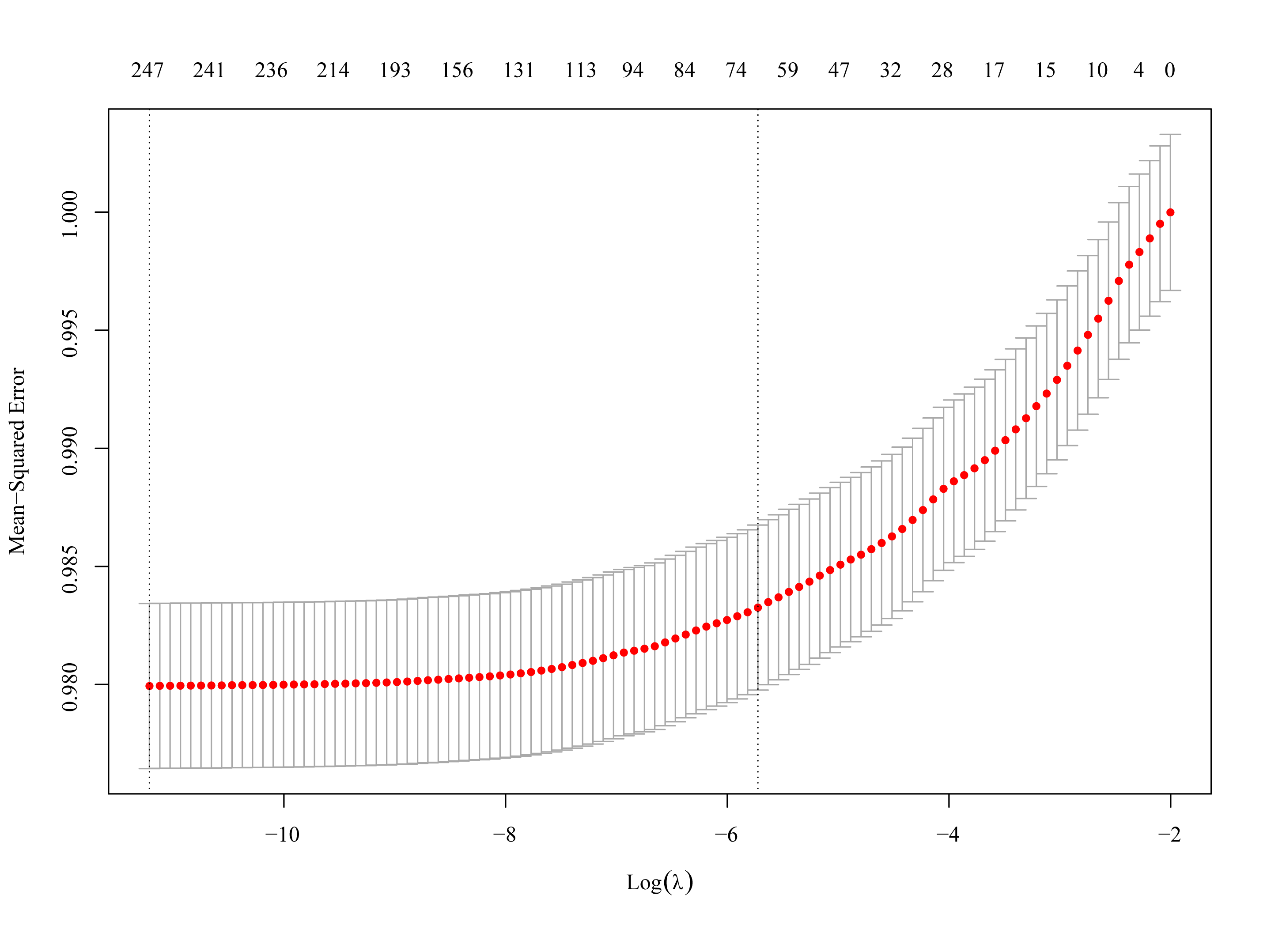


sFigure 3. Screening of metabolic signature for air pollution by elastic network regression.


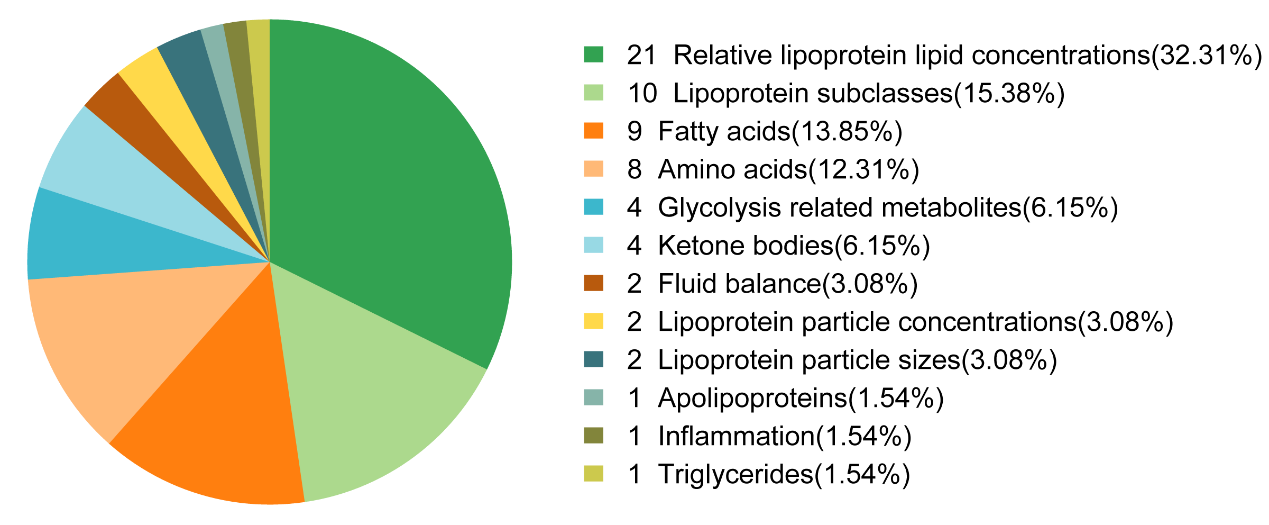


sFigure 4. Classification of 65 metabolic signature of air pollution screened by elastic network regression.


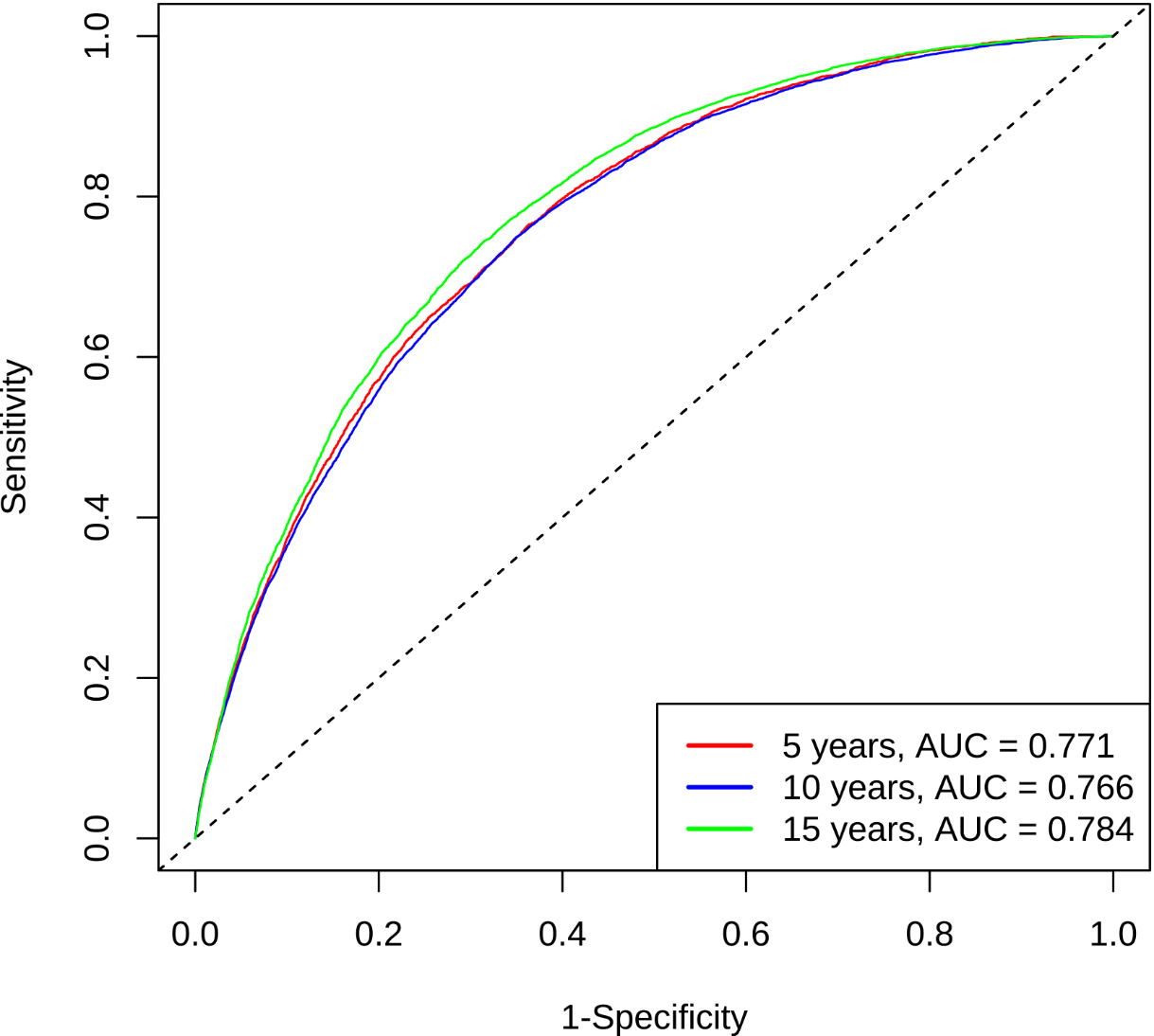


sFigure 5. ROC curve of air pollution related metabolic profiles predicting the risk of atrial fibrillation.

Model were adjusted for age, sex, race, BMI, physical activity, smoke, alcohol, DASH, history of diabetes mellitus, hypertension, CVD, and cancer. BMI: Body mass index; DASH: Dietary approaches to stop hypertension; CVD: Cardiovascular disease.

sTable 1. Description and group of metabolite by NMR in UK Biobank.

| No. | Field ID | Metabolite | Description | Group |
| --- | --- | --- | --- | --- |
| 1 | 23400 | Total_C | Total Cholesterol | Cholesterol |
| 2 | 23401 | non_HDL_C | Total Cholesterol Minus HDL-C | Cholesterol |
| 3 | 23402 | Remnant_C | Remnant Cholesterol (Non-HDL, Non-LDL -Cholesterol) | Cholesterol |
| 4 | 23403 | VLDL_C | VLDL Cholesterol | Cholesterol |
| 5 | 23404 | Clinical_LDL_C | Clinical LDL Cholesterol | Cholesterol |
| 6 | 23405 | LDL_C | LDL Cholesterol | Cholesterol |
| 7 | 23406 | HDL_C | HDL Cholesterol | Cholesterol |
| 8 | 23407 | Total_TG | Total Triglycerides | Triglycerides |
| 9 | 23408 | VLDL_TG | Triglycerides in VLDL | Triglycerides |
| 10 | 23409 | LDL_TG | Triglycerides in LDL | Triglycerides |
| 11 | 23410 | HDL_TG | Triglycerides in HDL | Triglycerides |
| 12 | 23411 | Total_PL | Total Phospholipids in Lipoprotein Particles | Phospholipids |
| 13 | 23412 | VLDL_PL | Phospholipids in VLDL | Phospholipids |
| 14 | 23413 | LDL_PL | Phospholipids in LDL | Phospholipids |
| 15 | 23414 | HDL_PL | Phospholipids in HDL | Phospholipids |
| 16 | 23415 | Total_CE | Total Esterified Cholesterol | Cholesteryl esters |
| 17 | 23416 | VLDL_CE | Cholesteryl Esters in VLDL | Cholesteryl esters |
| 18 | 23417 | LDL_CE | Cholesteryl Esters in LDL | Cholesteryl esters |
| 19 | 23418 | HDL_CE | Cholesteryl Esters in HDL | Cholesteryl esters |
| 20 | 23419 | Total_FC | Total Free Cholesterol | Free cholesterol |
| 21 | 23420 | VLDL_FC | Free Cholesterol in VLDL | Free cholesterol |
| 22 | 23421 | LDL_FC | Free Cholesterol in LDL | Free cholesterol |
| 23 | 23422 | HDL_FC | Free Cholesterol in HDL | Free cholesterol |
| 24 | 23423 | Total_L | Total Lipids in Lipoprotein Particles | Total lipids |
| 25 | 23424 | VLDL_L | Total Lipids in VLDL | Total lipids |
| 26 | 23425 | LDL_L | Total Lipids in LDL | Total lipids |
| 27 | 23426 | HDL_L | Total Lipids in HDL | Total lipids |
| 28 | 23427 | Total_P | Total Concentration of Lipoprotein Particles | Lipoprotein particle concentrations |
| 29 | 23428 | VLDL_P | Concentration of VLDL Particles | Lipoprotein particle concentrations |
| 30 | 23429 | LDL_P | Concentration of LDL Particles | Lipoprotein particle concentrations |
| 31 | 23430 | HDL_P | Concentration of HDL Particles | Lipoprotein particle concentrations |
| 32 | 23431 | VLDL_size | Average Diameter for VLDL Particles | Lipoprotein particle sizes |
| 33 | 23432 | LDL_size | Average Diameter for LDL Particles | Lipoprotein particle sizes |
| 34 | 23433 | HDL_size | Average Diameter for HDL Particles | Lipoprotein particle sizes |
| 35 | 23434 | Phosphoglyc | Phosphoglycerides | Other lipids |
| 36 | 23435 | TG_by_PG | Triglycerides to Phosphoglycerides ratio | Other lipids |
| 37 | 23436 | Cholines | Total Cholines | Other lipids |
| 38 | 23437 | Phosphatidylc | Phosphatidylcholines | Other lipids |
| 39 | 23438 | Sphingomyelins | Sphingomyelins | Other lipids |
| 40 | 23439 | ApoB | Apolipoprotein B | Apolipoproteins |
| 41 | 23440 | ApoA1 | Apolipoprotein A1 | Apolipoproteins |
| 42 | 23441 | ApoB_by_ApoA1 | Apolipoprotein B to Apolipoprotein A1 ratio | Apolipoproteins |
| 43 | 23442 | Total_FA | Total Fatty Acids | Fatty acids |
| 44 | 23443 | Unsaturation | Degree of Unsaturation | Fatty acids |
| 45 | 23444 | Omega_3 | Omega-3 Fatty Acids | Fatty acids |
| 46 | 23445 | Omega_6 | Omega-6 Fatty Acids | Fatty acids |
| 47 | 23446 | PUFA | Polyunsaturated Fatty Acids | Fatty acids |
| 48 | 23447 | MUFA | Monounsaturated Fatty Acids | Fatty acids |
| 49 | 23448 | SFA | Saturated Fatty Acids | Fatty acids |
| 50 | 23449 | LA | Linoleic Acid | Fatty acids |
| 51 | 23450 | DHA | Docosahexaenoic Acid | Fatty acids |
| 52 | 23451 | Omega_3_pct | Omega-3 Fatty Acids to Total Fatty Acids percentage | Fatty acids |
| 53 | 23452 | Omega_6_pct | Omega-6 Fatty Acids to Total Fatty Acids percentage | Fatty acids |
| 54 | 23453 | PUFA_pct | Polyunsaturated Fatty Acids to Total Fatty Acids percentage | Fatty acids |
| 55 | 23454 | MUFA_pct | Monounsaturated Fatty Acids to Total Fatty Acids percentage | Fatty acids |
| 56 | 23455 | SFA_pct | Saturated Fatty Acids to Total Fatty Acids percentage | Fatty acids |
| 57 | 23456 | LA_pct | Linoleic Acid to Total Fatty Acids percentage | Fatty acids |
| 58 | 23457 | DHA_pct | Docosahexaenoic Acid to Total Fatty Acids percentage | Fatty acids |
| 59 | 23458 | PUFA_by_MUFA | Polyunsaturated Fatty Acids to Monounsaturated Fatty Acids ratio | Fatty acids |
| 60 | 23459 | Omega_6_by_Omega_3 | Omega-6 Fatty Acids to Omega-3 Fatty Acids ratio | Fatty acids |
| 61 | 23460 | Ala | Alanine | Amino acids |
| 62 | 23461 | Gln | Glutamine | Amino acids |
| 63 | 23462 | Gly | Glycine | Amino acids |
| 64 | 23463 | His | Histidine | Amino acids |
| 65 | 23464 | Total_BCAA | Total Concentration of Branched-Chain Amino Acids (Leucine + Isoleucine + Valine) | Amino acids |
| 66 | 23465 | Ile | Isoleucine | Amino acids |
| 67 | 23466 | Leu | Leucine | Amino acids |
| 68 | 23467 | Val | Valine | Amino acids |
| 69 | 23468 | Phe | Phenylalanine | Amino acids |
| 70 | 23469 | Tyr | Tyrosine | Amino acids |
| 71 | 20281 | Ala_corrected | Spectrometer-corrected alanine | Amino acids |
| 72 | 23470 | Glucose | Glucose | Glycolysis related metabolites |
| 73 | 23471 | Lactate | Lactate | Glycolysis related metabolites |
| 74 | 23472 | Pyruvate | Pyruvate | Glycolysis related metabolites |
| 75 | 23473 | Citrate | Citrate | Glycolysis related metabolites |
| 76 | 20280 | Glucose_lactate | Glucose-lactate | Glycolysis related metabolites |
| 77 | 23474 | Hbutyrate | 3-Hydroxybutyrate | Ketone bodies |
| 78 | 23475 | Acetate | Acetate | Ketone bodies |
| 79 | 23476 | Acetoacetate | Acetoacetate | Ketone bodies |
| 80 | 23477 | Acetone | Acetone | Ketone bodies |
| 81 | 23478 | Creatinine | Creatinine | Fluid balance |
| 82 | 23479 | Albumin | Albumin | Fluid balance |
| 83 | 23480 | GlycA | Glycoprotein Acetyls | Inflammation |
| 84 | 23481 | XXL_VLDL_P | Concentration of Chylomicrons and Extremely Large VLDL Particles | Lipoprotein subclasses |
| 85 | 23482 | XXL_VLDL_L | Total Lipids in Chylomicrons and Extremely Large VLDL | Lipoprotein subclasses |
| 86 | 23483 | XXL_VLDL_PL | Phospholipids in Chylomicrons and Extremely Large VLDL | Lipoprotein subclasses |
| 87 | 23484 | XXL_VLDL_C | Cholesterol in Chylomicrons and Extremely Large VLDL | Lipoprotein subclasses |
| 88 | 23485 | XXL_VLDL_CE | Cholesteryl Esters in Chylomicrons and Extremely Large VLDL | Lipoprotein subclasses |
| 89 | 23486 | XXL_VLDL_FC | Free Cholesterol in Chylomicrons and Extremely Large VLDL | Lipoprotein subclasses |
| 90 | 23487 | XXL_VLDL_TG | Triglycerides in Chylomicrons and Extremely Large VLDL | Lipoprotein subclasses |
| 91 | 23488 | XL_VLDL_P | Concentration of Very Large VLDL Particles | Lipoprotein subclasses |
| 92 | 23489 | XL_VLDL_L | Total Lipids in Very Large VLDL | Lipoprotein subclasses |
| 93 | 23490 | XL_VLDL_PL | Phospholipids in Very Large VLDL | Lipoprotein subclasses |
| 94 | 23491 | XL_VLDL_C | Cholesterol in Very Large VLDL | Lipoprotein subclasses |
| 95 | 23492 | XL_VLDL_CE | Cholesteryl Esters in Very Large VLDL | Lipoprotein subclasses |
| 96 | 23493 | XL_VLDL_FC | Free Cholesterol in Very Large VLDL | Lipoprotein subclasses |
| 97 | 23494 | XL_VLDL_TG | Triglycerides in Very Large VLDL | Lipoprotein subclasses |
| 98 | 23495 | L_VLDL_P | Concentration of Large VLDL Particles | Lipoprotein subclasses |
| 99 | 23496 | L_VLDL_L | Total Lipids in Large VLDL | Lipoprotein subclasses |
| 100 | 23497 | L_VLDL_PL | Phospholipids in Large VLDL | Lipoprotein subclasses |
| 101 | 23498 | L_VLDL_C | Cholesterol in Large VLDL | Lipoprotein subclasses |
| 102 | 23499 | L_VLDL_CE | Cholesteryl Esters in Large VLDL | Lipoprotein subclasses |
| 103 | 23500 | L_VLDL_FC | Free Cholesterol in Large VLDL | Lipoprotein subclasses |
| 104 | 23501 | L_VLDL_TG | Triglycerides in Large VLDL | Lipoprotein subclasses |
| 105 | 23502 | M_VLDL_P | Concentration of Medium VLDL Particles | Lipoprotein subclasses |
| 106 | 23503 | M_VLDL_L | Total Lipids in Medium VLDL | Lipoprotein subclasses |
| 107 | 23504 | M_VLDL_PL | Phospholipids in Medium VLDL | Lipoprotein subclasses |
| 108 | 23505 | M_VLDL_C | Cholesterol in Medium VLDL | Lipoprotein subclasses |
| 109 | 23506 | M_VLDL_CE | Cholesteryl Esters in Medium VLDL | Lipoprotein subclasses |
| 110 | 23507 | M_VLDL_FC | Free Cholesterol in Medium VLDL | Lipoprotein subclasses |
| 111 | 23508 | M_VLDL_TG | Triglycerides in Medium VLDL | Lipoprotein subclasses |
| 112 | 23509 | S_VLDL_P | Concentration of Small VLDL Particles | Lipoprotein subclasses |
| 113 | 23510 | S_VLDL_L | Total Lipids in Small VLDL | Lipoprotein subclasses |
| 114 | 23511 | S_VLDL_PL | Phospholipids in Small VLDL | Lipoprotein subclasses |
| 115 | 23512 | S_VLDL_C | Cholesterol in Small VLDL | Lipoprotein subclasses |
| 116 | 23513 | S_VLDL_CE | Cholesteryl Esters in Small VLDL | Lipoprotein subclasses |
| 117 | 23514 | S_VLDL_FC | Free Cholesterol in Small VLDL | Lipoprotein subclasses |
| 118 | 23515 | S_VLDL_TG | Triglycerides in Small VLDL | Lipoprotein subclasses |
| 119 | 23516 | XS_VLDL_P | Concentration of Very Small VLDL Particles | Lipoprotein subclasses |
| 120 | 23517 | XS_VLDL_L | Total Lipids in Very Small VLDL | Lipoprotein subclasses |
| 121 | 23518 | XS_VLDL_PL | Phospholipids in Very Small VLDL | Lipoprotein subclasses |
| 122 | 23519 | XS_VLDL_C | Cholesterol in Very Small VLDL | Lipoprotein subclasses |
| 123 | 23520 | XS_VLDL_CE | Cholesteryl Esters in Very Small VLDL | Lipoprotein subclasses |
| 124 | 23521 | XS_VLDL_FC | Free Cholesterol in Very Small VLDL | Lipoprotein subclasses |
| 125 | 23522 | XS_VLDL_TG | Triglycerides in Very Small VLDL | Lipoprotein subclasses |
| 126 | 23523 | IDL_P | Concentration of IDL Particles | Lipoprotein subclasses |
| 127 | 23524 | IDL_L | Total Lipids in IDL | Lipoprotein subclasses |
| 128 | 23525 | IDL_PL | Phospholipids in IDL | Lipoprotein subclasses |
| 129 | 23526 | IDL_C | Cholesterol in IDL | Lipoprotein subclasses |
| 130 | 23527 | IDL_CE | Cholesteryl Esters in IDL | Lipoprotein subclasses |
| 131 | 23528 | IDL_FC | Free Cholesterol in IDL | Lipoprotein subclasses |
| 132 | 23529 | IDL_TG | Triglycerides in IDL | Lipoprotein subclasses |
| 133 | 23530 | L_LDL_P | Concentration of Large LDL Particles | Lipoprotein subclasses |
| 134 | 23531 | L_LDL_L | Total Lipids in Large LDL | Lipoprotein subclasses |
| 135 | 23532 | L_LDL_PL | Phospholipids in Large LDL | Lipoprotein subclasses |
| 136 | 23533 | L_LDL_C | Cholesterol in Large LDL | Lipoprotein subclasses |
| 137 | 23534 | L_LDL_CE | Cholesteryl Esters in Large LDL | Lipoprotein subclasses |
| 138 | 23535 | L_LDL_FC | Free Cholesterol in Large LDL | Lipoprotein subclasses |
| 139 | 23536 | L_LDL_TG | Triglycerides in Large LDL | Lipoprotein subclasses |
| 140 | 23537 | M_LDL_P | Concentration of Medium LDL Particles | Lipoprotein subclasses |
| 141 | 23538 | M_LDL_L | Total Lipids in Medium LDL | Lipoprotein subclasses |
| 142 | 23539 | M_LDL_PL | Phospholipids in Medium LDL | Lipoprotein subclasses |
| 143 | 23540 | M_LDL_C | Cholesterol in Medium LDL | Lipoprotein subclasses |
| 144 | 23541 | M_LDL_CE | Cholesteryl Esters in Medium LDL | Lipoprotein subclasses |
| 145 | 23542 | M_LDL_FC | Free Cholesterol in Medium LDL | Lipoprotein subclasses |
| 146 | 23543 | M_LDL_TG | Triglycerides in Medium LDL | Lipoprotein subclasses |
| 147 | 23544 | S_LDL_P | Concentration of Small LDL Particles | Lipoprotein subclasses |
| 148 | 23545 | S_LDL_L | Total Lipids in Small LDL | Lipoprotein subclasses |
| 149 | 23546 | S_LDL_PL | Phospholipids in Small LDL | Lipoprotein subclasses |
| 150 | 23547 | S_LDL_C | Cholesterol in Small LDL | Lipoprotein subclasses |
| 151 | 23548 | S_LDL_CE | Cholesteryl Esters in Small LDL | Lipoprotein subclasses |
| 152 | 23549 | S_LDL_FC | Free Cholesterol in Small LDL | Lipoprotein subclasses |
| 153 | 23550 | S_LDL_TG | Triglycerides in Small LDL | Lipoprotein subclasses |
| 154 | 23551 | XL_HDL_P | Concentration of Very Large HDL Particles | Lipoprotein subclasses |
| 155 | 23552 | XL_HDL_L | Total Lipids in Very Large HDL | Lipoprotein subclasses |
| 156 | 23553 | XL_HDL_PL | Phospholipids in Very Large HDL | Lipoprotein subclasses |
| 157 | 23554 | XL_HDL_C | Cholesterol in Very Large HDL | Lipoprotein subclasses |
| 158 | 23555 | XL_HDL_CE | Cholesteryl Esters in Very Large HDL | Lipoprotein subclasses |
| 159 | 23556 | XL_HDL_FC | Free Cholesterol in Very Large HDL | Lipoprotein subclasses |
| 160 | 23557 | XL_HDL_TG | Triglycerides in Very Large HDL | Lipoprotein subclasses |
| 161 | 23558 | L_HDL_P | Concentration of Large HDL Particles | Lipoprotein subclasses |
| 162 | 23559 | L_HDL_L | Total Lipids in Large HDL | Lipoprotein subclasses |
| 163 | 23560 | L_HDL_PL | Phospholipids in Large HDL | Lipoprotein subclasses |
| 164 | 23561 | L_HDL_C | Cholesterol in Large HDL | Lipoprotein subclasses |
| 165 | 23562 | L_HDL_CE | Cholesteryl Esters in Large HDL | Lipoprotein subclasses |
| 166 | 23563 | L_HDL_FC | Free Cholesterol in Large HDL | Lipoprotein subclasses |
| 167 | 23564 | L_HDL_TG | Triglycerides in Large HDL | Lipoprotein subclasses |
| 168 | 23565 | M_HDL_P | Concentration of Medium HDL Particles | Lipoprotein subclasses |
| 169 | 23566 | M_HDL_L | Total Lipids in Medium HDL | Lipoprotein subclasses |
| 170 | 23567 | M_HDL_PL | Phospholipids in Medium HDL | Lipoprotein subclasses |
| 171 | 23568 | M_HDL_C | Cholesterol in Medium HDL | Lipoprotein subclasses |
| 172 | 23569 | M_HDL_CE | Cholesteryl Esters in Medium HDL | Lipoprotein subclasses |
| 173 | 23570 | M_HDL_FC | Free Cholesterol in Medium HDL | Lipoprotein subclasses |
| 174 | 23571 | M_HDL_TG | Triglycerides in Medium HDL | Lipoprotein subclasses |
| 175 | 23572 | S_HDL_P | Concentration of Small HDL Particles | Lipoprotein subclasses |
| 176 | 23573 | S_HDL_L | Total Lipids in Small HDL | Lipoprotein subclasses |
| 177 | 23574 | S_HDL_PL | Phospholipids in Small HDL | Lipoprotein subclasses |
| 178 | 23575 | S_HDL_C | Cholesterol in Small HDL | Lipoprotein subclasses |
| 179 | 23576 | S_HDL_CE | Cholesteryl Esters in Small HDL | Lipoprotein subclasses |
| 180 | 23577 | S_HDL_FC | Free Cholesterol in Small HDL | Lipoprotein subclasses |
| 181 | 23578 | S_HDL_TG | Triglycerides in Small HDL | Lipoprotein subclasses |
| 182 | 23579 | XXL_VLDL_PL_pct | Phospholipids to Total Lipids in Chylomicrons and Extremely Large VLDL percentage | Relative lipoprotein lipid concentrations |
| 183 | 23580 | XXL_VLDL_C_pct | Cholesterol to Total Lipids in Chylomicrons and Extremely Large VLDL percentage | Relative lipoprotein lipid concentrations |
| 184 | 23581 | XXL_VLDL_CE_pct | Cholesteryl Esters to Total Lipids in Chylomicrons and Extremely Large VLDL percentage | Relative lipoprotein lipid concentrations |
| 185 | 23582 | XXL_VLDL_FC_pct | Free Cholesterol to Total Lipids in Chylomicrons and Extremely Large VLDL percentage | Relative lipoprotein lipid concentrations |
| 186 | 23583 | XXL_VLDL_TG_pct | Triglycerides to Total Lipids in Chylomicrons and Extremely Large VLDL percentage | Relative lipoprotein lipid concentrations |
| 187 | 23584 | XL_VLDL_PL_pct | Phospholipids to Total Lipids in Very Large VLDL percentage | Relative lipoprotein lipid concentrations |
| 188 | 23585 | XL_VLDL_C_pct | Cholesterol to Total Lipids in Very Large VLDL percentage | Relative lipoprotein lipid concentrations |
| 189 | 23586 | XL_VLDL_CE_pct | Cholesteryl Esters to Total Lipids in Very Large VLDL percentage | Relative lipoprotein lipid concentrations |
| 190 | 23587 | XL_VLDL_FC_pct | Free Cholesterol to Total Lipids in Very Large VLDL percentage | Relative lipoprotein lipid concentrations |
| 191 | 23588 | XL_VLDL_TG_pct | Triglycerides to Total Lipids in Very Large VLDL percentage | Relative lipoprotein lipid concentrations |
| 192 | 23589 | L_VLDL_PL_pct | Phospholipids to Total Lipids in Large VLDL percentage | Relative lipoprotein lipid concentrations |
| 193 | 23590 | L_VLDL_C_pct | Cholesterol to Total Lipids in Large VLDL percentage | Relative lipoprotein lipid concentrations |
| 194 | 23591 | L_VLDL_CE_pct | Cholesteryl Esters to Total Lipids in Large VLDL percentage | Relative lipoprotein lipid concentrations |
| 195 | 23592 | L_VLDL_FC_pct | Free Cholesterol to Total Lipids in Large VLDL percentage | Relative lipoprotein lipid concentrations |
| 196 | 23593 | L_VLDL_TG_pct | Triglycerides to Total Lipids in Large VLDL percentage | Relative lipoprotein lipid concentrations |
| 197 | 23594 | M_VLDL_PL_pct | Phospholipids to Total Lipids in Medium VLDL percentage | Relative lipoprotein lipid concentrations |
| 198 | 23595 | M_VLDL_C_pct | Cholesterol to Total Lipids in Medium VLDL percentage | Relative lipoprotein lipid concentrations |
| 199 | 23596 | M_VLDL_CE_pct | Cholesteryl Esters to Total Lipids in Medium VLDL percentage | Relative lipoprotein lipid concentrations |
| 200 | 23597 | M_VLDL_FC_pct | Free Cholesterol to Total Lipids in Medium VLDL percentage | Relative lipoprotein lipid concentrations |
| 201 | 23598 | M_VLDL_TG_pct | Triglycerides to Total Lipids in Medium VLDL percentage | Relative lipoprotein lipid concentrations |
| 202 | 23599 | S_VLDL_PL_pct | Phospholipids to Total Lipids in Small VLDL percentage | Relative lipoprotein lipid concentrations |
| 203 | 23600 | S_VLDL_C_pct | Cholesterol to Total Lipids in Small VLDL percentage | Relative lipoprotein lipid concentrations |
| 204 | 23601 | S_VLDL_CE_pct | Cholesteryl Esters to Total Lipids in Small VLDL percentage | Relative lipoprotein lipid concentrations |
| 205 | 23602 | S_VLDL_FC_pct | Free Cholesterol to Total Lipids in Small VLDL percentage | Relative lipoprotein lipid concentrations |
| 206 | 23603 | S_VLDL_TG_pct | Triglycerides to Total Lipids in Small VLDL percentage | Relative lipoprotein lipid concentrations |
| 207 | 23604 | XS_VLDL_PL_pct | Phospholipids to Total Lipids in Very Small VLDL percentage | Relative lipoprotein lipid concentrations |
| 208 | 23605 | XS_VLDL_C_pct | Cholesterol to Total Lipids in Very Small VLDL percentage | Relative lipoprotein lipid concentrations |
| 209 | 23606 | XS_VLDL_CE_pct | Cholesteryl Esters to Total Lipids in Very Small VLDL percentage | Relative lipoprotein lipid concentrations |
| 210 | 23607 | XS_VLDL_FC_pct | Free Cholesterol to Total Lipids in Very Small VLDL percentage | Relative lipoprotein lipid concentrations |
| 211 | 23608 | XS_VLDL_TG_pct | Triglycerides to Total Lipids in Very Small VLDL percentage | Relative lipoprotein lipid concentrations |
| 212 | 23609 | IDL_PL_pct | Phospholipids to Total Lipids in IDL percentage | Relative lipoprotein lipid concentrations |
| 213 | 23610 | IDL_C_pct | Cholesterol to Total Lipids in IDL percentage | Relative lipoprotein lipid concentrations |
| 214 | 23611 | IDL_CE_pct | Cholesteryl Esters to Total Lipids in IDL percentage | Relative lipoprotein lipid concentrations |
| 215 | 23612 | IDL_FC_pct | Free Cholesterol to Total Lipids in IDL percentage | Relative lipoprotein lipid concentrations |
| 216 | 23613 | IDL_TG_pct | Triglycerides to Total Lipids in IDL percentage | Relative lipoprotein lipid concentrations |
| 217 | 23614 | L_LDL_PL_pct | Phospholipids to Total Lipids in Large LDL percentage | Relative lipoprotein lipid concentrations |
| 218 | 23615 | L_LDL_C_pct | Cholesterol to Total Lipids in Large LDL percentage | Relative lipoprotein lipid concentrations |
| 219 | 23616 | L_LDL_CE_pct | Cholesteryl Esters to Total Lipids in Large LDL percentage | Relative lipoprotein lipid concentrations |
| 220 | 23617 | L_LDL_FC_pct | Free Cholesterol to Total Lipids in Large LDL percentage | Relative lipoprotein lipid concentrations |
| 221 | 23618 | L_LDL_TG_pct | Triglycerides to Total Lipids in Large LDL percentage | Relative lipoprotein lipid concentrations |
| 222 | 23619 | M_LDL_PL_pct | Phospholipids to Total Lipids in Medium LDL percentage | Relative lipoprotein lipid concentrations |
| 223 | 23620 | M_LDL_C_pct | Cholesterol to Total Lipids in Medium LDL percentage | Relative lipoprotein lipid concentrations |
| 224 | 23621 | M_LDL_CE_pct | Cholesteryl Esters to Total Lipids in Medium LDL percentage | Relative lipoprotein lipid concentrations |
| 225 | 23622 | M_LDL_FC_pct | Free Cholesterol to Total Lipids in Medium LDL percentage | Relative lipoprotein lipid concentrations |
| 226 | 23623 | M_LDL_TG_pct | Triglycerides to Total Lipids in Medium LDL percentage | Relative lipoprotein lipid concentrations |
| 227 | 23624 | S_LDL_PL_pct | Phospholipids to Total Lipids in Small LDL percentage | Relative lipoprotein lipid concentrations |
| 228 | 23625 | S_LDL_C_pct | Cholesterol to Total Lipids in Small LDL percentage | Relative lipoprotein lipid concentrations |
| 229 | 23626 | S_LDL_CE_pct | Cholesteryl Esters to Total Lipids in Small LDL percentage | Relative lipoprotein lipid concentrations |
| 230 | 23627 | S_LDL_FC_pct | Free Cholesterol to Total Lipids in Small LDL percentage | Relative lipoprotein lipid concentrations |
| 231 | 23628 | S_LDL_TG_pct | Triglycerides to Total Lipids in Small LDL percentage | Relative lipoprotein lipid concentrations |
| 232 | 23629 | XL_HDL_PL_pct | Phospholipids to Total Lipids in Very Large HDL percentage | Relative lipoprotein lipid concentrations |
| 233 | 23630 | XL_HDL_C_pct | Cholesterol to Total Lipids in Very Large HDL percentage | Relative lipoprotein lipid concentrations |
| 234 | 23631 | XL_HDL_CE_pct | Cholesteryl Esters to Total Lipids in Very Large HDL percentage | Relative lipoprotein lipid concentrations |
| 235 | 23632 | XL_HDL_FC_pct | Free Cholesterol to Total Lipids in Very Large HDL percentage | Relative lipoprotein lipid concentrations |
| 236 | 23633 | XL_HDL_TG_pct | Triglycerides to Total Lipids in Very Large HDL percentage | Relative lipoprotein lipid concentrations |
| 237 | 23634 | L_HDL_PL_pct | Phospholipids to Total Lipids in Large HDL percentage | Relative lipoprotein lipid concentrations |
| 238 | 23635 | L_HDL_C_pct | Cholesterol to Total Lipids in Large HDL percentage | Relative lipoprotein lipid concentrations |
| 239 | 23636 | L_HDL_CE_pct | Cholesteryl Esters to Total Lipids in Large HDL percentage | Relative lipoprotein lipid concentrations |
| 240 | 23637 | L_HDL_FC_pct | Free Cholesterol to Total Lipids in Large HDL percentage | Relative lipoprotein lipid concentrations |
| 241 | 23638 | L_HDL_TG_pct | Triglycerides to Total Lipids in Large HDL percentage | Relative lipoprotein lipid concentrations |
| 242 | 23639 | M_HDL_PL_pct | Phospholipids to Total Lipids in Medium HDL percentage | Relative lipoprotein lipid concentrations |
| 243 | 23640 | M_HDL_C_pct | Cholesterol to Total Lipids in Medium HDL percentage | Relative lipoprotein lipid concentrations |
| 244 | 23641 | M_HDL_CE_pct | Cholesteryl Esters to Total Lipids in Medium HDL percentage | Relative lipoprotein lipid concentrations |
| 245 | 23642 | M_HDL_FC_pct | Free Cholesterol to Total Lipids in Medium HDL percentage | Relative lipoprotein lipid concentrations |
| 246 | 23643 | M_HDL_TG_pct | Triglycerides to Total Lipids in Medium HDL percentage | Relative lipoprotein lipid concentrations |
| 247 | 23644 | S_HDL_PL_pct | Phospholipids to Total Lipids in Small HDL percentage | Relative lipoprotein lipid concentrations |
| 248 | 23645 | S_HDL_C_pct | Cholesterol to Total Lipids in Small HDL percentage | Relative lipoprotein lipid concentrations |
| 249 | 23646 | S_HDL_CE_pct | Cholesteryl Esters to Total Lipids in Small HDL percentage | Relative lipoprotein lipid concentrations |
| 250 | 23647 | S_HDL_FC_pct | Free Cholesterol to Total Lipids in Small HDL percentage | Relative lipoprotein lipid concentrations |
| 251 | 23648 | S_HDL_TG_pct | Triglycerides to Total Lipids in Small HDL percentage | Relative lipoprotein lipid concentrations |

sTable 2. Definitions of atrial fibrillation and other disease.

| **Disease** | **Field Name** | **Field ID** | **Data coding** | **Meaning** |
| --- | --- | --- | --- | --- |
| Atrial fibrillation | Non-cancer illness code, self-reported | 20002 | 1471;  1483. | Atrial fibrillation;  Atrial flutter. |
|  | Diagnoses - ICD 9 | 41271 | 4273 | Atrial fibrillation and flutter |
|  | Diagnoses - ICD10 | 41270 | I48;  I48.0;  I48.1;  I48.2;  I48.3;  I48.4;  I48.9. | Atrial fibrillation and flutter;  Paroxysmal atrial fibrillation;  Persistent atrial fibrillation;  Chronic atrial fibrillation;  Typical atrial flutter;  Atypical atrial flutter;  Atrial fibrillation and atrial flutter, unspecified. |
|  | Underlying (primary) cause of death: ICD10 | 40001 |  |  |
|  | Contributory (secondary) causes of death: ICD10 | 40002 |  |  |
|  | Operative procedures - OPCS4 | 41272 | K62.1;  K62.2;  K62.3;  K62.4. | Percutaneous transluminal ablation of pulmonary vein to left atrium conducting system;  Percutaneous transluminal ablation of atrial wall for atrial flutter;  Percutaneous transluminal ablation of conducting system of heart for atrial flutter NEC;  Percutaneous transluminal internal cardioversion NEC. |
| Diabetes mellitus | Non-cancer illness code, self-reported | 20002 | 1220;  1222;  1223. | Diabetes;  Type 1 diabetes;  Type 2 diabetes. |
|  | Diagnoses - ICD 9 | 41271 | 2500, 2501, 2502, 2503, 2504, 2505, 2509. | Diabetes mellitus |
|  | Diagnoses - ICD10 | 41270 | E10.0, E10.1, E10.2, E10.3, E10.4, E10.5, E10.6, E10.7, E10.8, E10.9;  E11.0, E11.1, E11.2, E11.3, E11.4, E11.5, E11.6, E11.7, E11.8, E11.9;  E12.1, E12.3, E12.5, E12.8, E12.9;  E13.0, E13.1, E13.2, E13.3, E13.4, E13.5, E13.6, E13.7, E13.8, E13.9;  E14.0, E14.1, E14.2, E14.3, E14.4, E14.5, E14.6, E14.7, E14.8, E14.9. | Insulin-dependent diabetes mellitus;  Non-insulin-dependent diabetes mellitus;  Malnutrition-related diabetes mellitus;  Other specified diabetes mellitus;  Unspecified diabetes mellitus. |
|  | Underlying (primary) cause of death: ICD10 | 40001 |  |  |
|  | Contributory (secondary) causes of death: ICD10 | 40002 |  |  |
|  | Medication for cholesterol, blood pressure, diabetes, or take exogenous hormones  (Sexed: Female only) | 6153 | 3 | Insulin |
|  | Medication for cholesterol, blood pressure or diabetes  (Sexed: Male only) | 6177 | 3 | Insulin |
|  | Treatment/medication code | 20003 | 1140857494, 1140857496, 1140857500, 1140857502, 1140857506, 1140857584, 1140857586, 1140857590, 1140868902, 1140868908, 1140874646, 1140874650, 1140874652, 1140874658, 1140874660, 1140874664, 1140874666, 1140874674, 1140874678, 1140874680, 1140874686, 1140874690, 1140874706, 1140874712, 1140874716, 1140874718, 1140874724, 1140874726, 1140874728, 1140874732, 1140874736, 1140874740, 1140874744, 1140874746, 1140882964, 1140883066, 1140884600, 1140910564, 1140910566, 1140910818, 1140921964, 1141152590, 1141153254, 1141153262, 1141156984, 1141157284, 1141168660, 1141168668, 1141169504, 1141171508, 1141171646, 1141171652, 1141173786, 1141173882, 1141177600, 1141177606, 1141189090, 1141189094. | Diabetes mellitus medication |
| Hypertension | Non-cancer Illness Codes | 20002 | 1065;  1072. | Hypertension;  Essential hypertension. |
|  | Diagnoses - ICD 9 | 41271 | 4010, 4011, 4019;  4039. | Essential hypertension;  Hypertensive renal disease. |
|  | Diagnoses – ICD 10 | 41270 | I10;  I11.X, I11.0, I11.9;  I12.X, I12.0, I12.9;  I13.X, I1.30, I13.1, I13.2, I13.9;  I15.0, I15.1, I15.2, I15.9. | Essential (primary) hypertension;  Hypertensive heart disease;  Hypertensive renal disease;  Hypertensive heart and renal disease;  Secondary hypertension. |
|  | Underlying (primary) cause of death: ICD10 | 40001 |  |  |
|  | Contributory (secondary) causes of death: ICD10 | 40002 |  |  |
|  | Medication for cholesterol, blood pressure, diabetes, or take exogenous hormones  (Sexed: Female only) | 6153 | 2 | Blood pressure medication |
|  | Medication for cholesterol, blood pressure or diabetes  (Sexed: Male only) | 6177 | 2 | Blood pressure medication |
|  | Treatment/medication code | 20003 | 1140860332, 1140860334, 1140860336, 1140860338, 1140860340, 1140860342, 1140860348, 1140860352, 1140860356, 1140860358, 1140860362, 1140860380, 1140860390, 1140860394, 1140860396, 1140860398, 1140860402, 1140860410, 1140860418, 1140860422, 1140860426, 1140860434, 1140860478, 1140860492, 1140860498, 1140860520, 1140860532, 1140860552, 1140860558, 1140860562, 1140860564, 1140860580, 1140860628, 1140860632, 1140860638, 1140860654, 1140860658, 1140860706, 1140860714, 1140860728, 1140860736, 1140860738, 1140860758, 1140860764, 1140860776, 1140860784, 1140860790, 1140860828, 1140860830, 1140860834, 1140860836, 1140860838, 1140860846, 1140860848, 1140860862, 1140860878, 1140860882, 1140860912, 1140860918, 1140860938, 1140860942, 1140860952, 1140860972, 1140860976, 1140860982, 1140860988, 1140860994, 1140861008, 1140861010, 1140861016, 1140861022, 1140861024, 1140861068, 1140861070, 1140861088, 1140861090, 1140861106, 1140861120, 1140861128, 1140861130, 1140861136, 1140861138, 1140861190, 1140861194, 1140861202, 1140861266, 1140861268, 1140861326, 1140861384, 1140864950, 1140864952, 1140866072, 1140866084, 1140866086, 1140866090, 1140866092, 1140866094, 1140866104, 1140866108, 1140866110, 1140866116, 1140866122, 1140866136, 1140866138, 1140866140, 1140866144, 1140866146, 1140866162, 1140866164, 1140866168, 1140866182, 1140866192, 1140866202, 1140866206, 1140866210, 1140866212, 1140866220, 1140866230, 1140866232, 1140866236, 1140866244, 1140866248, 1140866282, 1140866306, 1140866308, 1140866312, 1140866318, 1140866330, 1140866332, 1140866334, 1140866340, 1140866352, 1140866360, 1140866388, 1140866390, 1140866396, 1140866400, 1140866406, 1140866408, 1140866410, 1140866412, 1140866416, 1140866422, 1140866426, 1140866438, 1140866440, 1140866442, 1140866448, 1140866450, 1140866460, 1140866466, 1140866484, 1140866554, 1140866692, 1140866704, 1140866712, 1140866724, 1140866756, 1140866758, 1140866764, 1140866766, 1140866778, 1140866798, 1140866800, 1140866802, 1140866804, 1140875808, 1140879762, 1140879778, 1140879782, 1140879786, 1140879794, 1140879806, 1140879810, 1140879818, 1140879822, 1140879824, 1140879834, 1140879842, 1140879854, 1140879866, 1140888510, 1140888556, 1140888560, 1140888578, 1140888582, 1140888586, 1140888760, 1140888762, 1140909368, 1140911698, 1140916356, 1140923572, 1140923712, 1140923718, 1140926778, 1140926780, 1141145668, 1141151016, 1141151018, 1141151382, 1141152600, 1141153026, 1141153032, 1141153328, 1141156754, 1141156808, 1141157252, 1141157254, 1141164148, 1141164154, 1141164276, 1141165476, 1141166006, 1141167822, 1141167832, 1141171152, 1141172682, 1141172686, 1141172698, 1141173888, 1141180592, 1141187790, 1141190160, 1141192064, 1141193282, 1141193346, 1141194804, 1141194808, 1141194810, 1141201038, 1141201040, 1140860382, 1140860386, 1140860404, 1140860406, 1140860454, 1140860470, 1140860534, 1140860544, 1140860590, 1140860610, 1140860690, 1140860696, 1140860750, 1140860752, 1140860802, 1140860806, 1140860840, 1140860842, 1140860892, 1140860904, 1140860954, 1140860966, 1140861000, 1140861002, 1140861034, 1140861046, 1140861110, 1140861114, 1140861166, 1140861176, 1140861276, 1140861282, 1140866074, 1140866078, 1140866096, 1140866102, 1140866128, 1140866132, 1140866156, 1140866158, 1140866194, 1140866200, 1140866222, 1140866226, 1140866262, 1140866280, 1140866324, 1140866328, 1140866354, 1140866356, 1140866402, 1140866404, 1140866418, 1140866420, 1140866444, 1140866446, 1140866506, 1140866546, 1140866726, 1140866738, 1140866782, 1140866784, 1140879758, 1140879760, 1140879798, 1140879802, 1140879826, 1140879830, 1140888512, 1140888552, 1140888646, 1140888686, 1140916362, 1140917428, 1141145658, 1141145660, 1141152998, 1141153006, 1141156836, 1141156846, 1141164280, 1141165470, 1141171336, 1141171344, 1141180598, 1141187788, 1141194794, 1141194800. | Blood pressure medication |
| Ischemic heart disease | Non-cancer illness code, self-reported | 20002 | 1075 | Heart attack/myocardial infarction |
|  | Operation code, self-reported | 20004 | 1070;  1095;  1523. | Coronary angioplasty ± stent;  Coronary artery bypass grafts;  Triple heart bypass. |
|  | Diagnoses-ICD 9 | 41271 | 410;  411;  412;  413;  414. | Acute myocardial infarction;  Other acute and subacute forms of ischaemic heart disease;  Old myocardial infarction;  Angina pectoris;  Other forms of chronic ischaemic heart disease. |
|  | Diagnoses-ICD10 | 41270 | I21.X, I21.0, I21.1, I21.2, I21.3, I21.4, I21.9;  I22.X, I22.0, I22.1, I22.8, I22.9;  I23.X, I23.1, I23.2, I23.3, I23.6, I23.8;  I24.1;  I25.2. | Acute myocardial infarction;  Subsequent myocardial infarction;  Certain current complications following acute myocardial infarction;  Dressler’s syndrome;  Old myocardial infarction. |
|  | Underlying (primary) cause of death: ICD10 | 40001 |  |  |
|  | Contributory (secondary) causes of death: ICD10 | 40002 |  |  |
|  | Operative procedures-OPCS4 | 41272 | K40.1, K40.2, K40.3, K40.4;  K41.1, K41.2, K41.3, K41.4;  K45.1, K45.2, K45.3, K45.4, K45.5;  K49.1, K49.2, K49.8, K49.9;  K75.1, K75.2, K75.3, K75.4, K75.8, K75.9;  K50.2. | Saphenous vein graft replacement of (one – four or more) coronary artery;  Other autograft replacement of (one – four or more) coronary artery;  Connection of mammary/thoracic artery to the coronary artery;  Transluminal balloon angioplasty of coronary artery;  Percutaneous transluminal balloon angioplasty ± insertion of stent into the coronary artery;  Percutaneous transluminal coronary thrombolysis using streptokinase. |
| Heart failure | Non-cancer Illness Codes | 20002 | 1076;  1079. | Heart failure/pulmonary oedema;  Cardiomyopathy. |
|  | Diagnoses – ICD 9 | 41271 | 4254;  4280, 4281, 4289. | Other primary cardiomyopathies;  Heart failure. |
|  | Diagnoses – ICD 10 | 41270 | I11.0;  I13.0, I13.2;  I25.5;  I42.0, I42.5, I42.8, I42.9;  I50.0, I50.1, I50.9. | Hypertensive heart disease with (congestive) heart failure;  Hypertensive heart and renal disease with (congestive) heart failure and/or renal failure;  Ischaemic cardiomyopathy;  Dilated cardiomyopathy, Other restrictive/unspecified cardiomyopathies;  Heart failure. |
|  | Underlying (primary) cause of death: ICD10 | 40001 |  |  |
|  | Contributory (secondary) causes of death: ICD10 | 40002 |  |  |
| Stroke | Non-cancer illness code, self-reported | 20002 | 1081;  1491;  1583;  1086. | Stroke;  Brain haemorrhage;  Ischaemic stroke;  Subarachnoid haemorrhage. |
|  | Diagnoses – ICD 9 | 41271 | 4309;  4319;  4331, 4339;  4349. | Subarachnoid haemorrhage;  Intracerebral haemorrhage;  Occlusion and stenosis of precerebral arteries;  Occlusion of cerebral arteries. |
|  | Diagnoses – ICD 10 | 41270 | I60.0, I60.1, I60.2, I60.3, I60.4, I60.5, I60.6, I60.7, I60.8, I60.9;  I61.0, I61.1, I61.2, I61.3, I61.4, I61.5, I61.6, I61.7, I61.8, I61.9;  I621.0, I621.1, I62.9;  I63.0, I63.1, I63.2, I63.3, I63.4, I63.5, I63.6, I63.7, I63.8, I63.9;  I64. | Subarachnoid haemorrhage;  Intracerebral haemorrhage;  Other nontraumatic intracranial haemorrhage;  Cerebral infarction;  Stroke. |
|  | Underlying (primary) cause of death: ICD10 | 40001 |  |  |
|  | Contributory (secondary) causes of death: ICD10 | 40002 |  |  |
| Cancer | Cancer code, self-reported | 20001 | 1001~1088, -1, 99999. | All types of tumors |
|  | Cancer diagnosed by doctor | 2453 | 1 | Yes, a doctor has ever told you that you have had cancer |
|  | Diagnoses – ICD 9 | 41271 | 140~208 | Malignant neoplasm |
|  | Diagnoses – ICD 10 | 41270 | C00~C97 | Malignant neoplasm |
|  | Underlying (primary) cause of death: ICD10 | 40001 |  |  |
|  | Contributory (secondary) causes of death: ICD10 | 40002 |  |  |

sTable 3. Associations of air pollution components with atrial fibrillation.

| Exposure | | Model 1 | | | | Model 2 | | Model 3 | |
| --- | --- | --- | --- | --- | --- | --- | --- | --- | --- |
|  |  | HR (95%CI) | | *P* | | HR (95%CI) | *P* | HR (95%CI) | *P* |
| PM_2.5_ | | | | | | | | | |
| Each SD increment | 1.06 (1.04,1.07) | | <0.001 | | 1.03 (1.01,1.05) | | <0.001 | 1.02 (1.01,1.04) | 0.01 |
| Low | ref | |  | | ref | |  | ref |  |
| Medium | 1.06 (1.02,1.10) | | 0.01 | | 1.02 (0.98,1.06) | | 0.37 | 1.00 (0.96,1.04) | 0.93 |
| High | 1.18 (1.12,1.24) | | <0.001 | | 1.10 (1.04,1.15) | | <0.001 | 1.07 (1.02,1.12) | 0.01 |
| *P* for trend |  | | <0.001 | |  | | <0.001 |  | 0.01 |
| PM_10_ |  | |  | |  | |  |  |  |
| Each SD increment | 1.05 (1.04,1.07) | | <0.001 | | 1.04 (1.02,1.06) | | <0.001 | 1.03 (1.02,1.05) | <0.001 |
| Low | ref | |  | | ref | |  | ref |  |
| Medium | 1.09 (1.05,1.14) | | <0.001 | | 1.05 (1.01,1.09) | | 0.02 | 1.04 (1.00,1.08) | 0.04 |
| High | 1.20 (1.14,1.26) | | <0.001 | | 1.15 (1.09,1.21) | | <0.001 | 1.13 (1.08,1.19) | <0.001 |
| *P* for trend |  | | <0.001 | |  | | <0.001 |  | <0.001 |
| NO_2_ |  | |  | |  | |  |  |  |
| Each SD increment | 1.05 (1.04,1.07) | | <0.001 | | 1.04 (1.02,1.05) | | <0.001 | 1.03 (1.01,1.05) | <0.001 |
| Low | ref | |  | | ref | |  | ref |  |
| Medium | 1.09 (1.05,1.13) | | <0.001 | | 1.05 (1.01,1.09) | | 0.03 | 1.03 (0.99,1.07) | 0.18 |
| High | 1.18 (1.12,1.24) | | <0.001 | | 1.12 (1.07,1.18) | | <0.001 | 1.10 (1.04,1.15) | <0.001 |
| *P* for trend |  | | <0.001 | |  | | <0.001 |  | <0.001 |
| NO_x_ |  | |  | |  | |  |  |  |
| Each SD increment | 1.05 (1.04,1.07) | | <0.001 | | 1.03 (1.02,1.05) | | <0.001 | 1.02 (1.01,1.04) | 0.002 |
| Low | ref | |  | | ref | |  | ref |  |
| Medium | 1.09 (1.04,1.13) | | <0.001 | | 1.05 (1.01,1.09) | | 0.02 | 1.03 (0.99,1.07) | 0.17 |
| High | 1.19 (1.13,1.25) | | <0.001 | | 1.11 (1.06,1.17) | | <0.001 | 1.08 (1.03,1.14) | 0.001 |
| *P* for trend |  | | <0.001 | |  | | <0.001 |  | 0.001 |

Model 1 was adjusted for age, sex, and race;

Model 2 was adjusted for Model 1 + BMI, physical activity, smoke, alcohol, and DASH;

Model 3 was adjusted for Model 2 + history of diabetes mellitus, hypertension, CVD, and cancer.

BMI: Body mass index; DASH: Dietary approaches to stop hypertension; CVD: Cardiovascular disease.

sTable 4. Elastic net regression coefficients of the 65 metabolites included in the air pollution-related metabolic signature.

| No. | Metabolite | Coefficient | Group |
| --- | --- | --- | --- |
| 1 | Hbutyrate | -0.0213 | Ketone bodies |
| 2 | Acetate | 0.0134 | Ketone bodies |
| 3 | Acetoacetate | 0.0281 | Ketone bodies |
| 4 | Acetone | -0.0002 | Ketone bodies |
| 5 | Albumin | -0.0138 | Fluid balance |
| 6 | ApoB_by_ApoA1 | 0.0369 | Apolipoproteins |
| 7 | HDL_size | -0.0117 | Lipoprotein particle sizes |
| 8 | LDL_size | -0.0033 | Lipoprotein particle sizes |
| 9 | M_VLDL_C | -0.0432 | Lipoprotein subclasses |
| 10 | S_VLDL_C | -0.0369 | Lipoprotein subclasses |
| 11 | XXL_VLDL_C_pct | -0.0015 | Relative lipoprotein lipid concentrations |
| 12 | IDL_C_pct | -0.0206 | Relative lipoprotein lipid concentrations |
| 13 | M_LDL_C_pct | -0.004 | Relative lipoprotein lipid concentrations |
| 14 | XL_HDL_C_pct | 0.0107 | Relative lipoprotein lipid concentrations |
| 15 | L_VLDL_CE | -0.0057 | Lipoprotein subclasses |
| 16 | S_VLDL_CE | -0.0325 | Lipoprotein subclasses |
| 17 | L_LDL_CE_pct | -0.0076 | Relative lipoprotein lipid concentrations |
| 18 | M_LDL_CE_pct | -0.0271 | Relative lipoprotein lipid concentrations |
| 19 | M_VLDL_CE_pct | 0.0103 | Relative lipoprotein lipid concentrations |
| 20 | XL_VLDL_CE_pct | 0.0121 | Relative lipoprotein lipid concentrations |
| 21 | Citrate | -0.0231 | Glycolysis related metabolites |
| 22 | HDL_P | -0.0037 | Lipoprotein particle concentrations |
| 23 | L_LDL_P | -0.0089 | Lipoprotein subclasses |
| 24 | Creatinine | -0.0272 | Fluid balance |
| 25 | Unsaturation | 0.0768 | Fatty acids |
| 26 | XXL_VLDL_FC_pct | -0.0091 | Relative lipoprotein lipid concentrations |
| 27 | IDL_FC_pct | -0.0167 | Relative lipoprotein lipid concentrations |
| 28 | M_HDL_FC_pct | -0.0153 | Relative lipoprotein lipid concentrations |
| 29 | S_HDL_FC_pct | 0.0029 | Relative lipoprotein lipid concentrations |
| 30 | S_LDL_FC_pct | 0.0228 | Relative lipoprotein lipid concentrations |
| 31 | Glucose | 0.008 | Glycolysis related metabolites |
| 32 | Gln | -0.011 | Amino acids |
| 33 | Gly | 0.0044 | Amino acids |
| 34 | GlycA | 0.013 | Inflammation |
| 35 | His | 0.003 | Amino acids |
| 36 | Ile | 0.0183 | Amino acids |
| 37 | Lactate | -0.0248 | Glycolysis related metabolites |
| 38 | MUFA | 0.06 | Fatty acids |
| 39 | Omega_3 | -0.0209 | Fatty acids |
| 40 | Omega_3_pct | -0.022 | Fatty acids |
| 41 | Omega_6 | 0.0228 | Fatty acids |
| 42 | Omega_6_by_Omega_3 | 0.0528 | Fatty acids |
| 43 | Phe | -0.0067 | Amino acids |
| 44 | S_LDL_PL | -0.013 | Lipoprotein subclasses |
| 45 | XXL_VLDL_PL_pct | 0.0053 | Relative lipoprotein lipid concentrations |
| 46 | IDL_PL_pct | 0.0082 | Relative lipoprotein lipid concentrations |
| 47 | L_HDL_PL_pct | 0.0022 | Relative lipoprotein lipid concentrations |
| 48 | S_LDL_PL_pct | -0.043 | Relative lipoprotein lipid concentrations |
| 49 | S_VLDL_PL_pct | 0.005 | Relative lipoprotein lipid concentrations |
| 50 | XS_VLDL_PL_pct | 0.0008 | Relative lipoprotein lipid concentrations |
| 51 | PUFA_by_MUFA | 0.0415 | Fatty acids |
| 52 | Pyruvate | 0.0571 | Glycolysis related metabolites |
| 53 | SFA | 0.0185 | Fatty acids |
| 54 | SFA_pct | -0.0133 | Fatty acids |
| 55 | Ala_corrected | 0.0013 | Amino acids |
| 56 | Total_P | -0.0259 | Lipoprotein particle concentrations |
| 57 | IDL_TG | 0.0002 | Lipoprotein subclasses |
| 58 | LDL_TG | 0.0058 | Triglycerides |
| 59 | L_HDL_TG | -0.0365 | Lipoprotein subclasses |
| 60 | L_LDL_TG | 0.0708 | Lipoprotein subclasses |
| 61 | S_VLDL_TG | -0.0013 | Lipoprotein subclasses |
| 62 | M_LDL_TG_pct | 0.008 | Relative lipoprotein lipid concentrations |
| 63 | XL_HDL_TG_pct | -0.0028 | Relative lipoprotein lipid concentrations |
| 64 | Tyr | -0.0155 | Amino acids |
| 65 | Val | -0.0066 | Amino acids |

sTable 5. Median and Interquartile range of concentrations for the 65 metabolites quantified by NMR.

| No. | Metabolite | | Overall(n=227,324) | Participants without AF(n=211,089) | | Participants with AF(n=16,235) | |
| --- | --- | --- | --- | --- | --- | --- | --- |
| 1 | Hbutyrate | 0.043 [0.030,0.069] | | | 0.043 [0.029,0.068] | 0.046 [0.032,0.072] |  |
| 2 | Acetate | 0.015 [0.012,0.019] | | | 0.015 [0.012,0.019] | 0.015 [0.011,0.019] |  |
| 3 | Acetoacetate | 0.010 [0.006,0.016] | | | 0.010 [0.006,0.016] | 0.011 [0.007,0.017] |  |
| 4 | Acetone | 0.013 [0.011,0.016] | | | 0.013 [0.011,0.016] | 0.013 [0.011,0.016] |  |
| 5 | Albumin | 39.371 [37.301,41.441] | | | 39.418 [37.346,41.482] | 38.746 [36.677,40.853] |  |
| 6 | ApoB_by_ApoA1 | 0.586 [0.483,0.705] | | | 0.587 [0.484,0.707] | 0.571 [0.473,0.688] |  |
| 7 | HDL_size | 9.598 [9.484,9.752] | | | 9.600 [9.486,9.755] | 9.572 [9.468,9.712] |  |
| 8 | LDL_size | 23.935 [23.871,23.990] | | | 23.936 [23.873,23.991] | 23.919 [23.855,23.978] |  |
| 9 | M_VLDL_C | 0.169 [0.127,0.215] | | | 0.170 [0.129,0.216] | 0.154 [0.112,0.202] |  |
| 10 | S_VLDL_C | 0.156 [0.123,0.192] | | | 0.156 [0.124,0.192] | 0.149 [0.118,0.184] |  |
| 11 | XXL_VLDL_C_pct | 26.312 [23.029,31.935] | | | 26.354 [23.066,31.995] | 25.793 [22.575,31.226] |  |
| 12 | IDL_C_pct | 68.150 [66.114,69.751] | | | 68.200 [66.197,69.780] | 67.372 [65.022,69.277] |  |
| 13 | M_LDL_C_pct | 68.721 [67.708,69.448] | | | 68.744 [67.749,69.461] | 68.376 [67.142,69.251] |  |
| 14 | XL_HDL_C_pct | 49.392 [47.136,52.383] | | | 49.388 [47.140,52.378] | 49.446 [47.082,52.460] |  |
| 15 | L_VLDL_CE | 0.051 [0.036,0.068] | | | 0.051 [0.036,0.068] | 0.050 [0.036,0.065] |  |
| 16 | S_VLDL_CE | 0.097 [0.077,0.120] | | | 0.098 [0.077,0.121] | 0.094 [0.074,0.116] |  |
| 17 | L_LDL_CE_pct | 52.778 [51.969,53.493] | | | 52.791 [51.986,53.504] | 52.595 [51.750,53.350] |  |
| 18 | M_LDL_CE_pct | 49.309 [48.119,50.418] | | | 49.314 [48.122,50.423] | 49.240 [48.070,50.352] |  |
| 19 | M_VLDL_CE_pct | 16.393 [12.811,19.734] | | | 16.484 [12.932,19.800] | 15.102 [11.285,18.721] |  |
| 20 | XL_VLDL_CE_pct | 15.957 [12.959,19.812] | | | 16.031 [13.029,19.892] | 15.005 [12.162,18.672] |  |
| 21 | Citrate | 0.065 [0.057,0.073] | | | 0.064 [0.056,0.073] | 0.065 [0.057,0.074] |  |
| 22 | NA | 0.015 [0.014,0.017] | | | 0.015 [0.014,0.017] | 0.015 [0.013,0.016] |  |
| 23 | L_LDL_P | 0.001 [0.001,0.001] | | | 0.001 [0.001,0.001] | 0.001 [0.001,0.001] |  |
| 24 | Creatinine | 0.066 [0.058,0.075] | | | 0.066 [0.058,0.075] | 0.069 [0.061,0.079] |  |
| 25 | Unsaturation | 1.355 [1.304,1.405] | | | 1.356 [1.306,1.406] | 1.344 [1.292,1.396] |  |
| 26 | XXL_VLDL_FC_pct | 11.605 [10.469,13.719] | | | 11.614 [10.474,13.731] | 11.502 [10.390,13.558] |  |
| 27 | IDL_FC_pct | 17.789 [17.049,18.464] | | | 17.797 [17.061,18.467] | 17.677 [16.885,18.422] |  |
| 28 | M_HDL_FC_pct | 8.301 [7.863,8.740] | | | 8.312 [7.874,8.749] | 8.159 [7.713,8.610] |  |
| 29 | S_HDL_FC_pct | 9.863 [9.572,10.193] | | | 9.869 [9.578,10.198] | 9.787 [9.499,10.127] |  |
| 30 | S_LDL_FC_pct | 17.680 [16.383,18.652] | | | 17.700 [16.411,18.667] | 17.402 [16.039,18.436] |  |
| 31 | Glucose | 3.560 [3.119,4.033] | | | 3.554 [3.114,4.024] | 3.648 [3.190,4.158] |  |
| 32 | Gln | 0.551 [0.497,0.606] | | | 0.551 [0.497,0.606] | 0.552 [0.497,0.608] |  |
| 33 | Gly | 0.158 [0.128,0.201] | | | 0.159 [0.128,0.202] | 0.149 [0.121,0.184] |  |
| 34 | GlycA | 0.808 [0.734,0.889] | | | 0.807 [0.733,0.888] | 0.818 [0.745,0.900] |  |
| 35 | His | 0.065 [0.058,0.072] | | | 0.065 [0.059,0.072] | 0.064 [0.058,0.071] |  |
| 36 | Ile | 0.048 [0.039,0.060] | | | 0.048 [0.039,0.060] | 0.050 [0.040,0.062] |  |
| 37 | Lactate | 3.859 [3.189,4.619] | | | 3.857 [3.187,4.618] | 3.879 [3.211,4.636] |  |
| 38 | MUFA | 2.801 [2.358,3.365] | | | 2.800 [2.358,3.365] | 2.807 [2.361,3.367] |  |
| 39 | Omega_3 | 0.500 [0.380,0.648] | | | 0.501 [0.380,0.649] | 0.497 [0.379,0.642] |  |
| 40 | Omega_3_pct | 4.161 [3.341,5.138] | | | 4.157 [3.338,5.135] | 4.210 [3.385,5.173] |  |
| 41 | Omega_6 | 4.491 [4.063,4.954] | | | 4.502 [4.076,4.964] | 4.344 [3.905,4.805] |  |
| 42 | Omega_6_by_Omega_3 | 9.011 [7.173,11.445] | | | 9.030 [7.189,11.473] | 8.741 [6.980,11.081] |  |
| 43 | Phe | 0.046 [0.040,0.053] | | | 0.046 [0.040,0.053] | 0.048 [0.041,0.055] |  |
| 44 | S_LDL_PL | 0.088 [0.076,0.101] | | | 0.089 [0.077,0.101] | 0.085 [0.072,0.098] |  |
| 45 | XXL_VLDL_PL_pct | 15.764 [14.600,16.882] | | | 15.750 [14.577,16.866] | 15.947 [14.878,17.076] |  |
| 46 | IDL_PL_pct | 23.874 [23.269,24.457] | | | 23.867 [23.265,24.446] | 23.969 [23.317,24.596] |  |
| 47 | L_HDL_PL_pct | 50.073 [48.555,52.055] | | | 50.026 [48.525,51.980] | 50.770 [49.028,53.028] |  |
| 48 | S_LDL_PL_pct | 31.112 [30.080,32.216] | | | 31.104 [30.072,32.207] | 31.222 [30.192,32.330] |  |
| 49 | S_VLDL_PL_pct | 23.620 [22.312,24.928] | | | 23.659 [22.364,24.960] | 23.065 [21.694,24.432] |  |
| 50 | XS_VLDL_PL_pct | 29.227 [28.688,29.819] | | | 29.214 [28.678,29.801] | 29.410 [28.834,30.050] |  |
| 51 | PUFA_by_MUFA | 1.805 [1.572,2.021] | | | 1.810 [1.577,2.026] | 1.742 [1.510,1.963] |  |
| 52 | Pyruvate | 0.079 [0.063,0.096] | | | 0.079 [0.063,0.096] | 0.079 [0.064,0.096] |  |
| 53 | SFA | 4.024 [3.492,4.667] | | | 4.027 [3.496,4.670] | 3.990 [3.439,4.630] |  |
| 54 | SFA_pct | 33.890 [32.739,35.161] | | | 33.876 [32.729,35.141] | 34.070 [32.871,35.409] |  |
| 55 | Ala_corrected | 0.360 [0.314,0.412] | | | 0.360 [0.313,0.412] | 0.365 [0.319,0.417] |  |
| 56 | Total_P | 0.017 [0.015,0.018] | | | 0.017 [0.015,0.019] | 0.016 [0.015,0.018] |  |
| 57 | IDL_TG | 0.098 [0.083,0.116] | | | 0.098 [0.083,0.116] | 0.098 [0.085,0.116] |  |
| 58 | LDL_TG | 0.141 [0.119,0.169] | | | 0.141 [0.119,0.169] | 0.143 [0.121,0.170] |  |
| 59 | L_HDL_TG | 0.029 [0.022,0.037] | | | 0.029 [0.022,0.037] | 0.028 [0.022,0.037] |  |
| 60 | L_LDL_TG | 0.095 [0.081,0.112] | | | 0.095 [0.081,0.112] | 0.096 [0.082,0.113] |  |
| 61 | S_VLDL_TG | 0.157 [0.120,0.199] | | | 0.156 [0.120,0.199] | 0.160 [0.124,0.201] |  |
| 62 | M_LDL_TG_pct | 5.131 [4.442,6.102] | | | 5.106 [4.428,6.063] | 5.497 [4.678,6.632] |  |
| 63 | XL_HDL_TG_pct | 4.514 [3.150,6.566] | | | 4.489 [3.132,6.531] | 4.857 [3.395,7.032] |  |
| 64 | Tyr | 0.061 [0.053,0.071] | | | 0.061 [0.053,0.071] | 0.063 [0.055,0.074] |  |
| 65 | Val | 0.207 [0.181,0.236] | | | 0.206 [0.181,0.235] | 0.211 [0.185,0.240] |  |

sTable 6. Subgroup of the associations of air pollution score and the related metabolic profiles with atrial fibrillation.

| Subgroup | | Exposure | Metabolite Profiles of Air Pollutants | | | Air pollution score | | | | |
| --- | --- | --- | --- | --- | --- | --- | --- | --- | --- | --- |
|  |  | HR (95%CI) | *P* | *P*_-interaction_ | | HR (95%CI) | *P* | *P*_-interaction_ |  |  |
| Age | |  |  |  | <0.001 | |  |  | <0.001 |  |
| <65 year | |  |  |  |  | |  |  |  |  |
|  | | Each SD increment | 1.14 (0.96,1.36) | 0.14 |  | | 0.99 (0.97,1.01) | 0.40 |  |  |
|  | | Low | ref |  |  | | ref |  |  |  |
|  | | Medium | 1.06 (0.96,1.07) | <0.001 |  | | 1.00 (0.95,1.04) | 0.88 |  |  |
|  | | High | 0.85 (0.81,0.90) | <0.001 |  | | 1.00 (0.95,1.06) | 0.87 |  |  |
| ≥65 year | |  |  |  |  | |  |  |  |  |
|  | | Each SD increment | 1.22 (1.05,1.52) | 0.03 |  | | 1.03 (1.01,1.06) | 0.02 |  |  |
|  | | Low | ref |  |  | | ref |  |  |  |
|  | | Medium | 1.05 (0.99,1.10) | 0.11 |  | | 1.01 (0.96,1.07) | 0.59 |  |  |
|  | | High | 1.09 (1.01,1.16) | 0.02 |  | | 1.11 (1.03,1.19) | 0.003 |  |  |
| Sex | |  |  |  | 0.006 | |  |  | 0.018 |  |
| Female | |  |  |  |  | |  |  |  |  |
|  | | Each SD increment | 1.08 (0.91,1.28) | 0.39 |  | | 1.02 (1.00,1.04) | 0.05 |  |  |
|  | | Low | ref |  |  | | ref |  |  |  |
|  | | Medium | 1.00 (0.96,1.05) | 0.94 |  | | 1.00 (0.95,1.04) | 0.85 |  |  |
|  | | High | 1.04 (0.99,1.10) | 0.16 |  | | 1.09 (1.03,1.15) | 0.002 |  |  |
| Male | |  |  |  |  | |  |  |  |  |
|  | | Each SD increment | 1.43 (1.14,1.81) | 0.002 |  | | 1.05 (1.02,1.08) | <0.001 |  |  |
|  | | Low | ref |  |  | | ref |  |  |  |
|  | | Medium | 1.08 (1.02,1.14) | 0.01 |  | | 1.10 (1.04,1.17) | <0.001 |  |  |
|  | | High | 1.10 (1.02,1.18) | 0.02 |  | | 1.14 (1.06,1.22) | <0.001 |  |  |
| Race | |  |  |  | 0.049 | |  |  | 0.047 |  |
| White | |  |  |  |  | |  |  |  |  |
|  | | Each SD increment | 1.21 (1.06,1.4) | 0.006 |  | | 1.03 (1.01,1.05) | <0.001 |  |  |
|  | | Low |  |  |  | |  |  |  |  |
|  | | Medium | 1.06 (1.02,1.1) | 0.002 |  | | 1.06 (1.02,1.1) | 0.001 |  |  |
|  | | High | 1.12 (1.07,1.17) | <0.001 |  | | 1.14 (1.09,1.19) | <0.001 |  |  |
| Other | |  |  |  |  | |  |  |  |  |
|  | | Each SD increment | 0.44 (0.22,1.19) | 0.125 |  | | 1.02 (0.93,1.11) | 0.732 |  |  |
|  | | Low |  |  |  | |  |  |  |  |
|  | | Medium | 1 (0.74,1.36) | 0.977 |  | | 1.27 (0.91,1.77) | 0.163 |  |  |
|  | | High | 0.93 (0.7,1.24) | 0.62 |  | | 1.28 (0.93,1.75) | 0.133 |  |  |

Models were adjusted for age, sex, race, BMI, physical activity, smoke, alcohol, DASH, history of diabetes mellitus, hypertension, CVD, and cancer. BMI: Body mass index; DASH: Dietary approaches to stop hypertension; CVD: Cardiovascular disease.

sTable 7. Cox regression model to assess associations between 65 metabolites of air pollution and atrial fibrillation.

| No. | Metabolite | HR (95% CI) | *P* |
| --- | --- | --- | --- |
| 1 | Hbutyrate | 1.04 (1.03,1.06) | <0.001 |
| 2 | Acetate | 1.01 (1.00,1.02) | 0.04 |
| 3 | Acetoacetate | 1.04 (1.03,1.06) | <0.001 |
| 4 | Acetone | 1.06 (1.05,1.07) | <0.001 |
| 5 | Albumin | 0.91 (0.90,0.93) | <0.001 |
| 6 | ApoB_by_ApoA1 | 0.93 (0.91,0.94) | <0.001 |
| 7 | HDL_size | 1.14 (1.12,1.16) | <0.001 |
| 8 | LDL_size | 1.05 (1.04,1.07) | <0.001 |
| 9 | M_VLDL_C | 0.92 (0.91,0.94) | <0.001 |
| 10 | S_VLDL_C | 0.91 (0.90,0.93) | <0.001 |
| 11 | XXL_VLDL_C_pct | 1.05 (1.03,1.06) | <0.001 |
| 12 | IDL_C_pct | 1.02 (1.00,1.03) | 0.06 |
| 13 | M_LDL_C_pct | 0.98 (0.97,1.00) | 0.02 |
| 14 | XL_HDL_C_pct | 0.96 (0.94,0.97) | <0.001 |
| 15 | L_VLDL_CE | 0.89 (0.87,0.90) | <0.001 |
| 16 | S_VLDL_CE | 0.91 (0.90,0.93) | <0.001 |
| 17 | L_LDL_CE_pct | 0.96 (0.95,0.97) | <0.001 |
| 18 | M_LDL_CE_pct | 0.92 (0.91,0.93) | <0.001 |
| 19 | M_VLDL_CE_pct | 1.10 (1.08,1.12) | <0.001 |
| 20 | XL_VLDL_CE_pct | 1.09 (1.07,1.11) | <0.001 |
| 21 | Citrate | 1.02 (1.00,1.03) | 0.06 |
| 22 | HDL_P | 0.99 (0.97,1.01) | 0.22 |
| 23 | L_LDL_P | 0.93 (0.91,0.94) | <0.001 |
| 24 | Creatinine | 1.01 (0.99,1.02) | 0.39 |
| 25 | Unsaturation | 1.01 (0.99,1.03) | 0.19 |
| 26 | XXL_VLDL_FC_pct | 1.06 (1.04,1.08) | <0.001 |
| 27 | IDL_FC_pct | 1.04 (1.03,1.06) | <0.001 |
| 28 | M_HDL_FC_pct | 1.05 (1.03,1.07) | <0.001 |
| 29 | S_HDL_FC_pct | 1.04 (1.02,1.06) | <0.001 |
| 30 | S_LDL_FC_pct | 1.04 (1.03,1.06) | <0.001 |
| 31 | Glucose | 0.98 (0.97,1.00) | 0.01 |
| 32 | Gln | 1.01 (0.99,1.02) | 0.27 |
| 33 | Gly | 1.00 (0.98,1.01) | 0.63 |
| 34 | GlycA | 0.94 (0.93,0.96) | <0.001 |
| 35 | His | 0.94 (0.92,0.95) | <0.001 |
| 36 | Ile | 0.96 (0.94,0.97) | <0.001 |
| 37 | Lactate | 0.99 (0.97,1.00) | 0.13 |
| 38 | MUFA | 0.92 (0.91,0.94) | <0.001 |
| 39 | Omega_3 | 0.93 (0.92,0.95) | <0.001 |
| 40 | Omega_3_pct | 0.97 (0.95,0.98) | <0.001 |
| 41 | Omega_6 | 0.92 (0.90,0.93) | <0.001 |
| 42 | Omega_6_by_Omega_3 | 1.04 (1.03,1.06) | <0.001 |
| 43 | Phe | 1.02 (1.00,1.03) | 0.04 |
| 44 | S_LDL_PL | 0.92 (0.91,0.94) | <0.001 |
| 45 | XXL_VLDL_PL_pct | 1.00 (0.98,1.02) | 0.9 |
| 46 | IDL_PL_pct | 1.03 (1.01,1.04) | <0.001 |
| 47 | L_HDL_PL_pct | 0.94 (0.92,0.95) | <0.001 |
| 48 | S_LDL_PL_pct | 1.09 (1.07,1.10) | <0.001 |
| 49 | S_VLDL_PL_pct | 1.05 (1.03,1.07) | <0.001 |
| 50 | XS_VLDL_PL_pct | 1.01 (0.99,1.02) | 0.34 |
| 51 | PUFA_by_MUFA | 1.07 (1.05,1.08) | <0.001 |
| 52 | Pyruvate | 1.00 (0.99,1.02) | 0.83 |
| 53 | SFA | 0.93 (0.92,0.95) | <0.001 |
| 54 | SFA_pct | 1.00 (0.98,1.01) | 0.8 |
| 55 | Ala_corrected | 0.95 (0.94,0.97) | <0.001 |
| 56 | Total_P | 0.98 (0.96,0.99) | 0.01 |
| 57 | IDL_TG | 0.95 (0.93,0.97) | <0.001 |
| 58 | LDL_TG | 0.93 (0.92,0.95) | <0.001 |
| 59 | L_HDL_TG | 0.99 (0.98,1.01) | 0.45 |
| 60 | L_LDL_TG | 0.94 (0.93,0.96) | <0.001 |
| 61 | S_VLDL_TG | 0.90 (0.88,0.91) | <0.001 |
| 62 | M_LDL_TG_pct | 1.00 (0.99,1.02) | 0.65 |
| 63 | XL_HDL_TG_pct | 0.92 (0.91,0.94) | <0.001 |
| 64 | Tyr | 1.01 (1.00,1.03) | 0.07 |
| 65 | Val | 0.94 (0.92,0.95) | <0.001 |

Models were adjusted for age, sex, race, BMI, physical activity, smoke, alcohol, DASH, history of diabetes mellitus, hypertension, CVD, and cancer. BMI: Body mass index; DASH: Dietary approaches to stop hypertension; CVD: Cardiovascular disease.

sTable 8. Mediation of metabolic signature on the association of air pollution score and components with atrial fibrillation.

| Exposure | HR (95%CI) | *P* |
| --- | --- | --- |
| Air pollution |  |  |
| Direct effect | 1.10 (1.07,1.13) | <0.001 |
| Indirect effect | 1.02 (1.01,1.02) | <0.001 |
| Total effect | 1.12 (1.09,1.15) | <0.001 |
| Mediation proportion (%) | 15.45 (11.84,20.77) | <0.001 |
| PM_2.5_ |  |  |
| Direct effect | 1.06 (1.03,1.09) | <0.001 |
| Indirect effect | 1.01 (1.01,1.02) | <0.001 |
| Total effect | 1.07 (1.04,1.11) | <0.001 |
| Mediation proportion (%) | 20.52 (13.29,32.99) | <0.001 |
| PM_10_ |  |  |
| Direct effect | 1.07 (1.04,1.11) | <0.001 |
| Indirect effect | 1.01 (1.01,1.02) | <0.001 |
| Total effect | 1.09 (1.06,1.12) | <0.001 |
| Mediation proportion (%) | 17.50 (12.21,26.89) | <0.001 |
| NO_2_ |  |  |
| Direct effect | 1.08 (1.05,1.11) | <0.001 |
| Indirect effect | 1.01 (1.01,1.02) | <0.001 |
| Total effect | 1.09 (1.06,1.12) | <0.001 |
| Mediation proportion (%) | 16.50 (12.79,23.73) | <0.001 |
| NO_x_ |  |  |
| Direct effect | 1.09 (1.06,1.11) | <0.001 |
| Indirect effect | 1.01 (1.01,1.02) | <0.001 |
| Total effect | 1.11 (1.07,1.13) | <0.001 |
| Mediation proportion (%) | 15.13 (12.09,20.74) | <0.001 |

Models were adjusted for age, sex, race, BMI, physical activity, smoke, alcohol, DASH, history of diabetes mellitus, hypertension, CVD, and cancer. BMI: Body mass index; DASH: Dietary approaches to stop hypertension; CVD: Cardiovascular disease.

sTable 9. Mediation proportion of 65 air pollution metabolites on the association of air pollution score with atrial fibrillation.

| Metabolite | Mediation proportion (%) | *P* |
| --- | --- | --- |
| Hbutyrate | 0.57 (0.39,1.25) | <0.001 |
| Acetate | -0.01 (-0.11,0.12) | <0.001 |
| Acetoacetate | 1.68 (1.23,2.76) | <0.001 |
| Acetone | 0.85 (0.42,1.49) | <0.001 |
| Albumin | 9.48 (8.58,13.53) | <0.001 |
| ApoB_by_ApoA1 | 1.45 (0.86,2.56) | <0.001 |
| HDL_size | 1.65 (1.08,2.18) | <0.001 |
| LDL_size | 0.96 (0.57,1.78) | <0.001 |
| M_VLDL_C | 10.69 (9.10,14.68) | <0.001 |
| S_VLDL_C | 5.22 (4.14,7.33) | <0.001 |
| XXL_VLDL_C_pct | 0.18 (0.01,0.29) | <0.001 |
| IDL_C_pct | 8.14 (6.84,11.29) | <0.001 |
| M_LDL_C_pct | 5.95 (5.08,6.97) | <0.001 |
| XL_HDL_C_pct | -0.17 (-0.52,0.05) | 0.212 |
| L_VLDL_CE | 2.33 (1.62,3.28) | 0.252 |
| S_VLDL_CE | 3.89 (3.01,5.52) | 0.921 |
| L_LDL_CE_pct | 3.16 (2.55,3.74) | 0.610 |
| M_LDL_CE_pct | 1.57 (1.25,2.03) | <0.001 |
| M_VLDL_CE_pct | 4.35 (3.97,5.43) | 0.410 |
| XL_VLDL_CE_pct | 1.50 (1.15,2.21) | 0.645 |
| Citrate | -0.30 (-0.75,-0.11) | <0.001 |
| HDL_P | 5.35 (4.51,7.30) | <0.001 |
| L_LDL_P | 9.22 (7.57,12.94) | <0.001 |
| Creatinine | -0.97 (-1.64,-0.70) | <0.001 |
| Unsaturation | 6.27 (5.19,8.75) | <0.001 |
| XXL_VLDL_FC_pct | -0.22 (-0.47,-0.08) | <0.001 |
| IDL_FC_pct | 1.96 (1.70,2.37) | <0.001 |
| M_HDL_FC_pct | 9.37 (8.49,12.22) | 0.221 |
| S_HDL_FC_pct | 6.59 (6.09,8.33) | <0.001 |
| S_LDL_FC_pct | 2.22 (1.70,3.08) | <0.001 |
| Glucose | 0.93 (0.79,1.31) | <0.001 |
| Gln | 1.36 (1.03,1.63) | <0.001 |
| Gly | 0.46 (0.04,0.82) | 0.292 |
| GlycA | 2.11 (1.63,3.33) | <0.001 |
| His | 2.24 (1.98,3.47) | <0.001 |
| Ile | -0.07 (-0.27,0.10) | <0.001 |
| Lactate | 0.04 (-0.03,0.22) | <0.001 |
| MUFA | -0.38 (-0.60,-0.31) | 0.473 |
| Omega_3 | 7.47 (5.21,10.98) | 0.856 |
| Omega_3_pct | 4.25 (2.76,6.40) | <0.001 |
| Omega_6 | 8.15 (6.57,11.19) | 0.941 |
| Omega_6_by_Omega_3 | 2.43 (1.53,3.88) | <0.001 |
| Phe | -0.20 (-0.44,-0.03) | <0.001 |
| S_LDL_PL | 7.99 (6.65,11.48) | <0.001 |
| XXL_VLDL_PL_pct | 1.91 (1.65,2.72) | <0.001 |
| IDL_PL_pct | 2.87 (2.01,4.26) | <0.001 |
| L_HDL_PL_pct | 5.62 (4.86,7.25) | <0.001 |
| S_LDL_PL_pct | 0.80 (0.10,1.31) | 0.837 |
| S_VLDL_PL_pct | 5.74 (5.19,7.65) | <0.001 |
| XS_VLDL_PL_pct | 9.21 (7.87,11.83) | <0.001 |
| PUFA_by_MUFA | 4.82 (4.33,6.81) | <0.001 |
| Pyruvate | 1.34 (0.61,2.24) | <0.001 |
| SFA | 1.72 (1.29,2.73) | <0.001 |
| SFA_pct | -0.68 (-1.10,-0.26) | <0.001 |
| Ala_corrected | -0.31 (-0.55,-0.11) | <0.001 |
| Total_P | 7.40 (6.30,9.89) | <0.001 |
| IDL_TG | 0.09 (-0.03,0.23) | <0.001 |
| LDL_TG | -0.02 (-0.14,0.08) | <0.001 |
| L_HDL_TG | 1.01 (0.54,1.90) | <0.001 |
| L_LDL_TG | 0.07 (-0.06,0.21) | <0.001 |
| S_VLDL_TG | -0.09 (-0.26,0.00) | <0.001 |
| M_LDL_TG_pct | 5.51 (4.90,6.83) | <0.001 |
| XL_HDL_TG_pct | 0.40 (-0.02,0.53) | <0.001 |
| Tyr | -1.62 (-2.28,-1.39) | <0.001 |
| Val | -0.40 (-0.84,0.02) | <0.001 |

Models were adjusted for age, sex, race, BMI, physical activity, smoke, alcohol, DASH, history of diabetes mellitus, hypertension, CVD, and cancer. BMI: Body mass index; DASH: Dietary approaches to stop hypertension; CVD: Cardiovascular disease.

sTable 10. Sensitive analysis of the associations of air pollution score and the related metabolic profiles with atrial fibrillation after exclusion of participants with missing covariates.

| Exposure | Model 1 | | Model 2 | | | Model 3 | | | |  |
| --- | --- | --- | --- | --- | --- | --- | --- | --- | --- | --- |
|  | HR (95%CI) | *P* | HR (95%CI) | *P* | | HR (95%CI) | | *P* | |  |
| Metabolite Profiles of Air Pollutants | | | | | | | | | |  |
| Each SD increment | 2.26 (1.93,2.65) | <0.001 | 1.47 (1.25,1.73) | | <0.001 | | 1.12 (1.05,1.32) | | 0.019 | |
| Low | ref |  | ref | |  | | ref | |  | |
| Medium | 1.14 (1.10,1.19) | <0.001 | 1.07 (1.03,1.12) | | <0.001 | | 1.03 (0.99,1.08) | | 0.152 | |
| High | 1.27 (1.21,1.34) | <0.001 | 1.13 (1.07,1.19) | | <0.001 | | 1.08 (1.02,1.11) | | 0.030 | |
| *P* for trend |  | <0.001 |  | | <0.001 | |  | | 0.010 | |
| Air pollution score |  |  |  | |  | |  | |  | |
| Each SD increment | 1.05 (1.03,1.07) | <0.001 | 1.03 (1.01,1.05) | | 0.002 | | 1.02 (1.00,1.04) | | 0.011 | |
| Low | ref |  | ref | |  | | ref | |  | |
| Medium | 1.07 (1.03,1.12) | <0.001 | 1.03 (0.99,1.08) | | 0.110 | | 1.03 (0.98,1.07) | | 0.240 | |
| High | 1.15 (1.10,1.22) | <0.001 | 1.10 (1.05,1.16) | | <0.001 | | 1.09 (1.04,1.15) | | <0.001 | |
| *P* for trend |  | <0.001 |  | <0.001 | |  | | 0.002 | |  |

The sample was 164,376

Model 1 was adjusted for age, sex, and race;

Model 2 was adjusted for Model 1 + BMI, physical activity, smoke, alcohol, and DASH;

Model 3 was adjusted for Model 2 + history of diabetes mellitus, hypertension, CVD, and cancer.

BMI: Body mass index; DASH: Dietary approaches to stop hypertension; CVD: Cardiovascular disease.

sTable 11. Sensitive analysis of the mediation of metabolic signature on the association of air pollution score and components with atrial fibrillation after exclusion of participants with missing covariates.

| Exposure | HR (95%CI) | *P* |
| --- | --- | --- |
| Air pollution |  |  |
| Direct effect | 1.08 (1.06,1.11) | <0.001 |
| Indirect effect | 1.01 (1.01,1.02) | <0.001 |
| Total effect | 1.10 (1.07,1.13) | <0.001 |
| Mediation proportion (%) | 15.12 (12.01,19.50) | <0.001 |
| PM_2.5_ |  |  |
| Direct effect | 1.05 (1.01,1.08) | <0.001 |
| Indirect effect | 1.01 (1.01,1.02) | <0.001 |
| Total effect | 1.06 (1.02,1.10) | <0.001 |
| Mediation proportion (%) | 21.60 (14.46,76.97) | <0.001 |
| PM_10_ |  |  |
| Direct effect | 1.08 (1.06,1.12) | <0.001 |
| Indirect effect | 1.01 (1.01,1.02) | <0.001 |
| Total effect | 1.09 (1.07,1.13) | <0.001 |
| Mediation proportion (%) | 15.19 (11.52,20.67) | <0.001 |
| NO_2_ |  |  |
| Direct effect | 1.07 (1.04,1.10) | <0.001 |
| Indirect effect | 1.01 (1.01,1.02) | <0.001 |
| Total effect | 1.09 (1.05,1.11) | <0.001 |
| Mediation proportion (%) | 16.25 (11.15,27.12) | <0.001 |
| NO_x_ |  |  |
| Direct effect | 1.07 (1.03,1.10) | <0.001 |
| Indirect effect | 1.01 (1.01,1.02) | <0.001 |
| Total effect | 1.08 (1.04,1.11) | <0.001 |
| Mediation proportion (%) | 17.62 (13.23,32.73) | <0.001 |

The sample was 164,376

Models were adjusted for age, sex, race, BMI, physical activity, smoke, alcohol, DASH, history of diabetes mellitus, hypertension, CVD, and cancer. BMI: Body mass index; DASH: Dietary approaches to stop hypertension; CVD: Cardiovascular disease.

sTable 12. Sensitive analysis of the associations of air pollution score and the related metabolic profiles with atrial fibrillation after exclusion of participants who developed atrial fibrillation within 2 years of follow-up.

| Exposure | Model 1 | | Model 2 | | | Model 3 | | | |  |
| --- | --- | --- | --- | --- | --- | --- | --- | --- | --- | --- |
|  | HR (95%CI) | *P* | HR (95%CI) | *P* | | HR (95%CI) | | *P* | |  |
| Metabolite Profiles of Air Pollutants | | | | | | | | | |  |
| Each SD increment | 2.45 (2.15,2.80) | <0.001 | 1.65 (1.43,1.89) | | <0.001 | | 1.22 (1.06,1.41) | | 0.010 | |
| Low | ref |  | ref | |  | | ref | |  | |
| Medium | 1.16 (1.12,1.20) | <0.001 | 1.08 (1.05,1.12) | | <0.001 | | 1.04 (1.00,1.07) | | 0.060 | |
| High | 1.32 (1.26,1.38) | <0.001 | 1.17 (1.12,1.22) | | <0.001 | | 1.07 (1.02,1.12) | | 0.004 | |
| *P* for trend |  | <0.001 |  | | <0.001 | |  | | 0.003 | |
| Air pollution score |  |  |  | |  | |  | |  | |
| Each SD increment | 1.06 (1.04,1.08) | <0.001 | 1.04 (1.02,1.06) | | <0.001 | | 1.03 (1.02,1.05) | | <0.001 | |
| Low | ref |  | ref | |  | | ref | |  | |
| Medium | 1.09 (1.05,1.13) | <0.001 | 1.05 (1.01,1.09) | | 0.01 | | 1.04 (1.00,1.07) | | 0.052 | |
| High | 1.19 (1.14,1.24) | <0.001 | 1.13 (1.08,1.18) | | <0.001 | | 1.11 (1.06,1.16) | | <0.001 | |
| *P* for trend |  | <0.001 |  | | <0.001 | |  | | <0.001 | |

The sample was 226,200

Model 1 was adjusted for age, sex, and race;

Model 2 was adjusted for Model 1 + BMI, physical activity, smoke, alcohol, and DASH;

Model 3 was adjusted for Model 2 + history of diabetes mellitus, hypertension, CVD, and cancer.

BMI: Body mass index; DASH: Dietary approaches to stop hypertension; CVD: Cardiovascular disease.

sTable 13. Sensitive analysis of the mediation of metabolic signature on the association of air pollution score and components with atrial fibrillation after exclusion of participants who developed atrial fibrillation within 2 years of follow-up.

| Exposure | HR (95%CI) | *P* |
| --- | --- | --- |
| Air pollution |  |  |
| Direct effect | 1.10 (1.07,1.11) | <0.001 |
| Indirect effect | 1.02 (1.01,1.02) | <0.001 |
| Total effect | 1.12 (1.09,1.13) | <0.001 |
| Mediation proportion (%) | 14.92 (12.58,21.28) | <0.001 |
| PM_2.5_ |  |  |
| Direct effect | 1.07 (1.04,1.11) | <0.001 |
| Indirect effect | 1.01 (1.01,1.02) | <0.001 |
| Total effect | 1.08 (1.06,1.13) | <0.001 |
| Mediation proportion (%) | 18.93 (12.27,29.48) | <0.001 |
| PM_10_ |  |  |
| Direct effect | 1.10 (1.07,1.13) | <0.001 |
| Indirect effect | 1.02 (1.01,1.02) | <0.001 |
| Total effect | 1.12 (1.09,1.14) | <0.001 |
| Mediation proportion (%) | 14.76 (11.85,19.90) | <0.001 |
| NO_2_ |  |  |
| Direct effect | 1.09 (1.06,1.10) | <0.001 |
| Indirect effect | 1.02 (1.01,1.02) | <0.001 |
| Total effect | 1.11 (1.08,1.12) | <0.001 |
| Mediation proportion (%) | 15.71 (14.31,22.74) | <0.001 |
| NO_x_ |  |  |
| Direct effect | 1.09 (1.07,1.12) | <0.001 |
| Indirect effect | 1.02 (1.01,1.02) | <0.001 |
| Total effect | 1.11 (1.08,1.14) | <0.001 |
| Mediation proportion (%) | 15.31 (11.06,21.46) | <0.001 |

The sample was 226,200

Models were adjusted for age, sex, race, BMI, physical activity, smoke, alcohol, DASH, history of diabetes mellitus, hypertension, CVD, and cancer. BMI: Body mass index; DASH: Dietary approaches to stop hypertension; CVD: Cardiovascular disease.

sTable 14. Sensitive analysis of the associations of air pollution score and the related metabolic profiles with atrial fibrillation after exclusion of participants who has the history of chronic disease.

| Exposure | Model 1 | | Model 2 | | |  |
| --- | --- | --- | --- | --- | --- | --- |
|  | HR (95%CI) | *P* | HR (95%CI) | *P* | |  |
| Metabolite Profiles of Air Pollutants | | | | | |  |
| Each SD increment | 1.62 (1.29,2.05) | <0.001 | 1.33 (1.05,1.68) | | 0.02 | |
| Low | ref |  | ref | |  | |
| Medium | 1.10 (1.04,1.17) | <0.001 | 1.07 (1.01,1.14) | | 0.02 | |
| High | 1.14 (1.05,1.23) | 0.001 | 1.08 (1.00,1.18) | | 0.05 | |
| *P* for trend |  | <0.001 |  | | 0.01 | |
| Air pollution score |  |  |  | |  | |
| Each SD increment | 1.04 (1.01,1.07) | 0.004 | 1.03 (1.00,1.06) | | 0.05 | |
| Low | ref |  | ref | |  | |
| Medium | 1.05 (0.99,1.11) | 0.13 | 1.02 (0.97,1.09) | | 0.43 | |
| High | 1.13 (1.05,1.22) | 0.001 | 1.10 (1.02,1.18) | | 0.01 | |
| *P* for trend |  | 0.002 |  | | 0.02 | |

The sample was 138,613

Model 1 was adjusted for age, sex, and race;

Model 2 was adjusted for Model 1 + BMI, physical activity, smoke, alcohol, and DASH.

BMI: Body mass index; DASH: Dietary approaches to stop hypertension.

sTable 15. Sensitive analysis of the mediation of metabolic signature on the association of air pollution score and components with atrial fibrillation after exclusion of participants who has the history of chronic disease.

| Exposure | HR (95%CI) | *P* |
| --- | --- | --- |
| Air pollution |  |  |
| Direct effect | 1.06 (1.01,1.11) | <0.001 |
| Indirect effect | 1.01 (1.00,1.01) | <0.001 |
| Total effect | 1.07 (1.02,1.12) | <0.001 |
| Mediation proportion (%) | 11.17 (6.25,17.06) | <0.001 |
| PM_2.5_ |  |  |
| Direct effect | 1.02 (0.97,1.05) | <0.001 |
| Indirect effect | 1.01 (1.00,1.01) | <0.001 |
| Total effect | 1.03 (1.01,1.06) | <0.001 |
| Mediation proportion (%) | 15.82 (6.85,21.82) | <0.001 |
| PM_10_ |  |  |
| Direct effect | 1.10 (1.03,1.20) | <0.001 |
| Indirect effect | 1.01 (1.00,1.01) | <0.001 |
| Total effect | 1.11 (1.03,1.20) | <0.001 |
| Mediation proportion (%) | 7.11 (4.46,23.11) | <0.001 |
| NO_2_ |  |  |
| Direct effect | 1.07 (1.00,1.12) | <0.001 |
| Indirect effect | 1.01 (1.00,1.01) | <0.001 |
| Total effect | 1.07 (1.01,1.13) | <0.001 |
| Mediation proportion (%) | 10.43 (5.32,18.25) | <0.001 |
| NO_x_ |  |  |
| Direct effect | 1.05 (0.99,1.12) | <0.001 |
| Indirect effect | 1.01 (1.00,1.01) | <0.001 |
| Total effect | 1.05 (1.01,1.13) | <0.001 |
| Mediation proportion (%) | 14.09 (9.31,21.21) | <0.001 |

The sample was 138,613

Models were adjusted for age, sex, race, BMI, physical activity, smoke, alcohol, and DASH. BMI: Body mass index; DASH: Dietary approaches to stop hypertension.

sTable 16. Sensitive analysis of the associations of air pollution score and the related metabolic profiles with atrial fibrillation after excluding participants who were taking a statin at baseline.

| Exposure | Model 1 | | Model 2 | | | Model 3 | | | |  |
| --- | --- | --- | --- | --- | --- | --- | --- | --- | --- | --- |
|  | HR (95%CI) | *P* | HR (95%CI) | *P* | | HR (95%CI) | | *P* | |  |
| Metabolite Profiles of Air Pollutants | | | | | | | | | |  |
| Each SD increment | 1.78 (1.51,2.10) | <0.001 | 1.37 (1.15,1.62) | | <0.001 | | 1.27 (1.07,1.50) | | 0.01 | |
| Low | ref |  | ref | |  | | ref | |  | |
| Medium | 1.11 (1.06,1.15) | <0.001 | 1.06 (1.01,1.10) | | 0.01 | | 1.04 (1.00,1.09) | | 0.05 | |
| High | 1.17 (1.11,1.24) | <0.001 | 1.09 (1.03,1.16) | | 0.002 | | 1.07 (1.01,1.13) | | 0.02 | |
| *P* for trend |  | <0.001 |  | | <0.001 | |  | | 0.01 | |
| Air pollution score |  |  |  | |  | |  | |  | |
| Each SD increment | 1.05 (1.03,1.07) | <0.001 | 1.03 (1.01,1.05) | | <0.001 | | 1.03 (1.01,1.05) | | 0.01 | |
| Low | ref |  | ref | |  | | ref | |  | |
| Medium | 1.07 (1.03,1.12) | 0.001 | 1.04 (1.00,1.08) | | 0.07 | | 1.03 (0.99,1.08) | | 0.13 | |
| High | 1.14 (1.08,1.21) | <0.001 | 1.11 (1.05,1.17) | | <0.001 | | 1.09 (1.03,1.15) | | 0.001 | |
| *P* for trend |  | <0.001 |  | | <0.001 | |  | | 0.002 | |

The sample was 187,052

Model 1 was adjusted for age, sex, and race;

Model 2 was adjusted for Model 1 + BMI, physical activity, smoke, alcohol, and DASH;

Model 3 was adjusted for Model 2 + history of diabetes mellitus, hypertension, CVD, and cancer.

BMI: Body mass index; DASH: Dietary approaches to stop hypertension; CVD: Cardiovascular disease.

sTable 17. Sensitive analysis of the mediation of metabolic signature on the association of air pollution score and components with atrial fibrillation after excluding participants who were taking a statin at baseline.

| Exposure | HR (95%CI) | *P* |
| --- | --- | --- |
| Air pollution |  |  |
| Direct effect | 1.09 (1.05,1.14) | <0.001 |
| Indirect effect | 1.08 (1.05,1.13) | <0.001 |
| Total effect | 1.01 (1.01,1.01) | <0.001 |
| Mediation proportion (%) | 11.01 (7,18.12) | <0.001 |
| PM2.5 |  |  |
| Direct effect | 1.06 (1.02,1.09) | <0.001 |
| Indirect effect | 1.05 (1.01,1.08) | <0.001 |
| Total effect | 1.01 (1.01,1.01) | <0.001 |
| Mediation proportion (%) | 16.15 (10.11,44.85) | <0.001 |
| PM10 |  |  |
| Direct effect | 1.11 (1.05,1.13) | <0.001 |
| Indirect effect | 1.1 (1.04,1.12) | <0.001 |
| Total effect | 1.01 (1.01,1.01) | <0.001 |
| Mediation proportion (%) | 9.59 (6.63,23.24) | <0.001 |
| NO2 |  |  |
| Direct effect | 1.08 (1.05,1.13) | <0.001 |
| Indirect effect | 1.07 (1.04,1.12) | <0.001 |
| Total effect | 1.01 (1.01,1.01) | <0.001 |
| Mediation proportion (%) |  |  |
| NOx | 11.47 (7.32,15.31) | <0.001 |
| Direct effect | 1.09 (1.07,1.13) | <0.001 |
| Indirect effect | 1.08 (1.07,1.12) | <0.001 |
| Total effect | 1.01 (1.01,1.01) | <0.001 |
| Mediation proportion (%) | 11.07 (7.37,13.46) | <0.001 |

The sample was 187,052

Models were adjusted for age, sex, race, BMI, physical activity, smoke, alcohol, DASH, history of diabetes mellitus, hypertension, CVD, and cancer. BMI: Body mass index; DASH: Dietary approaches to stop hypertension; CVD: Cardiovascular disease.

sTable 18. Sensitive analysis of the associations of air pollution score and the related metabolic profiles with atrial fibrillation after using the 2010 air pollution concentrations.

| Exposure | Model 1 | | Model 2 | | | Model 3 | | | |  |
| --- | --- | --- | --- | --- | --- | --- | --- | --- | --- | --- |
|  | HR (95%CI) | *P* | HR (95%CI) | *P* | | HR (95%CI) | | *P* | |  |
| Metabolite Profiles of Air Pollutants | | | | | | | | | |  |
| Each SD increment | 3.17 (2.79,3.60) | <0.001 | 1.68 (1.46,1.93) | | <0.001 | | 1.18 (1.02,1.36) | | 0.03 | |
| Low | ref |  | ref | |  | | ref | |  | |
| Medium | 1.18 (1.14,1.22) | <0.001 | 1.07 (1.03,1.10) | | <0.001 | | 1.01 (0.98,1.05) | | 0.44 | |
| High | 1.42 (1.36,1.48) | <0.001 | 1.17 (1.12,1.23) | | <0.001 | | 1.06 (1.01,1.11) | | 0.01 | |
| *P* for trend |  | <0.001 |  | | <0.001 | |  | | 0.01 | |
| Air pollution score |  |  |  | |  | |  | |  | |
| Each SD increment | 1.06 (1.04,1.07) | <0.001 | 1.03 (1.02,1.05) | | <0.001 | | 1.02 (1.01,1.04) | | 0.003 | |
| Low | ref |  | ref | |  | | ref | |  | |
| Medium | 1.05 (1.02,1.09) | 0.003 | 1.02 (0.98,1.05) | | 0.34 | | 1.01 (0.97,1.04) | | 0.78 | |
| High | 1.16 (1.11,1.21) | <0.001 | 1.09 (1.05,1.14) | | <0.001 | | 1.07 (1.03,1.12) | | 0.001 | |
| *P* for trend |  | <0.001 |  | | <0.001 | |  | | 0.004 | |

Model 1 was adjusted for age, sex, and race;

Model 2 was adjusted for Model 1 + BMI, physical activity, smoke, alcohol, and DASH;

Model 3 was adjusted for Model 2 + history of diabetes mellitus, hypertension, CVD, and cancer.

BMI: Body mass index; DASH: Dietary approaches to stop hypertension; CVD: Cardiovascular disease.

sTable 19. Sensitive analysis of the mediation of metabolic signature on the association of air pollution score and components with atrial fibrillation after using the 2010 air pollution concentrations.

| Exposure | HR (95%CI) | *P* |
| --- | --- | --- |
| Air pollution |  |  |
| Direct effect | 1.08 (1.05,1.09) | <0.001 |
| Indirect effect | 1.06 (1.03,1.07) | <0.001 |
| Total effect | 1.02 (1.02,1.02) | <0.001 |
| Mediation proportion (%) | 28.69 (26.41,50.29) | <0.001 |
| PM_2.5_ |  |  |
| Direct effect | 1.08 (1.06,1.12) | <0.001 |
| Indirect effect | 1.06 (1.03,1.1) | <0.001 |
| Total effect | 1.02 (1.02,1.03) | <0.001 |
| Mediation proportion (%) | 30.89 (20.54,48.81) | <0.001 |
| PM_10_ |  |  |
| Direct effect | 1.07 (1.05,1.1) | <0.001 |
| Indirect effect | 1.06 (1.03,1.08) | <0.001 |
| Total effect | 1.02 (1.01,1.02) | <0.001 |
| Mediation proportion (%) | 21.84 (16.78,33.64) | <0.001 |
| NO_2_ |  |  |
| Direct effect | 1.1 (1.06,1.12) | <0.001 |
| Indirect effect | 1.07 (1.04,1.1) | <0.001 |
| Total effect | 1.02 (1.02,1.03) | <0.001 |
| Mediation proportion (%) | 26.44 (20.52,40.14) | <0.001 |
| NO_x_ |  |  |
| Direct effect | 1.11 (1.08,1.14) | <0.001 |
| Indirect effect | 1.08 (1.06,1.11) | <0.001 |
| Total effect | 1.02 (1.02,1.03) | <0.001 |
| Mediation proportion (%) | 24.38 (18.47,33.98) | <0.001 |

Models were adjusted for age, sex, race, BMI, physical activity, smoke, alcohol, DASH, history of diabetes mellitus, hypertension, CVD, and cancer. BMI: Body mass index; DASH: Dietary approaches to stop hypertension; CVD: Cardiovascular disease.

sTable 20. Sensitive analysis of the associations of air pollution score and the related metabolic profiles with atrial fibrillation after further adjusting for traffic noise, availability of green space, and aspirin use.

| Exposure | HR (95%CI) | *P* |
| --- | --- | --- |
| Metabolite Profiles of Air Pollutants | | |
| Each SD increment | 1.17(1.02,1.35) | 0.03 |
| Low | ref |  |
| Medium | 1.02(0.99,1.06) | 0.22 |
| High | 1.06(1.01,1.11) | 0.01 |
| *P* for trend |  | 0.01 |
| Air pollution score |  |  |
| Each SD increment | 1.05(1.03,1.08) | <0.001 |
| Low | ref |  |
| Medium | 1.06(1.01,1.10) | 0.01 |
| High | 1.15(1.09,1.22) | <0.001 |
| *P* for trend |  | <0.001 |

Models was adjusted for age, sex, and race, BMI, physical activity, smoke, alcohol, DASH, history of diabetes mellitus, hypertension, CVD, and cancer, traffic noise, availability of green space, and aspirin. BMI: Body mass index; DASH: Dietary approaches to stop hypertension; CVD: Cardiovascular disease.

sTable 21. Sensitive analysis of the mediation of metabolic signature on the association of air pollution score and components with atrial fibrillation after further adjusting for traffic noise, availability of green space, and aspirin use.

| Exposure | HR (95%CI) | *P* |
| --- | --- | --- |
| Air pollution |  |  |
| Direct effect | 1.11(1.07,1.19) | <0.001 |
| Indirect effect | 1.10(1.06,1.18) | <0.001 |
| Total effect | 1.01(1.01,1.01) | <0.001 |
| Mediation proportion (%) | 10.64(7.6,15.19) | <0.001 |
| PM_2.5_ |  |  |
| Direct effect | 1.03(1.01,1.10) | <0.001 |
| Indirect effect | 1.02(1.00,1.01) | <0.001 |
| Total effect | 1.01(1.00,1.00) | <0.001 |
| Mediation proportion (%) | 18.83(8.38,29.14) | <0.001 |
| PM_10_ |  |  |
| Direct effect | 1.01(1.06,1.12) | <0.001 |
| Indirect effect | 1.09(1.05,1.10) | <0.001 |
| Total effect | 1.01(1.01,1.01) | <0.001 |
| Mediation proportion (%) | 10.67(9.31,19.03) | <0.001 |
| NO_2_ |  |  |
| Direct effect | 1.10(1.07,1.15) | <0.001 |
| Indirect effect | 1.09(1.06,1.13) | <0.001 |
| Total effect | 1.01(1.01,1.01) | <0.001 |
| Mediation proportion (%) | 11.79(8.18,12.67) | <0.001 |
| NO_x_ |  |  |
| Direct effect | 1.10(1.07,1.16) | <0.001 |
| Indirect effect | 1.09(1.06,1.15) | <0.001 |
| Total effect | 1.01(1.01,1.01) | <0.001 |
| Mediation proportion (%) | 9.72(6.46,10.76) | <0.001 |

Models was adjusted for age, sex, and race, BMI, physical activity, smoke, alcohol, DASH, history of diabetes mellitus, hypertension, CVD, and cancer, traffic noise, availability of green space, and aspirin. BMI: Body mass index; DASH: Dietary approaches to stop hypertension; CVD: Cardiovascular disease.

sTable 22. Sensitive analysis of the associations of air pollution score and the related metabolic profiles with atrial fibrillation after using PCA to construct the air pollution score.

| Exposure | Model 1 | | Model 2 | | | Model 3 | | | |  |
| --- | --- | --- | --- | --- | --- | --- | --- | --- | --- | --- |
|  | HR (95%CI) | *P* | HR (95%CI) | *P* | | HR (95%CI) | | *P* | |  |
| Metabolite Profiles of Air Pollutants | | | | | | | | | |  |
| Each SD increment | 2.90(2.54,3.31) | <0.001 | 1.69(1.47,1.95) | | <0.001 | | 1.19(1.03,1.38) | | 0.02 | |
| Low | ref |  | ref | |  | | ref | |  | |
| Medium | 1.17(1.13,1.21) | <0.001 | 1.08(1.04,1.12) | | <0.001 | | 1.03(0.99,1.07) | | 0.09 | |
| High | 1.39(1.33,1.45) | <0.001 | 1.19(1.14,1.24) | | <0.001 | | 1.08(1.03,1.13) | | <0.001 | |
| *P* for trend |  | <0.001 |  | | <0.001 | |  | | 0.001 | |
| Air pollution score |  |  |  | |  | |  | |  | |
| Each SD increment | 1.06(1.04,1.08) | <0.001 | 1.04(1.02,1.06) | | <0.001 | | 1.03(1.01,1.05) | | <0.001 | |
| Low | ref |  | ref | |  | | ref | |  | |
| Medium | 1.08(1.04,1.13) | <0.001 | 1.04(1.00,1.08) | | 0.05 | | 1.02(0.98,1.07) | | 0.25 | |
| High | 1.20(1.14,1.26) | <0.001 | 1.12(1.07,1.18) | | <0.001 | | 1.10(1.04,1.15) | | <0.001 | |
| *P* for trend |  | <0.001 |  | | <0.001 | |  | | <0.001 | |

Model 1 was adjusted for age, sex, and race;

Model 2 was adjusted for Model 1 + BMI, physical activity, smoke, alcohol, and DASH;

Model 3 was adjusted for Model 2 + history of diabetes mellitus, hypertension, CVD, and cancer.

BMI: Body mass index; DASH: Dietary approaches to stop hypertension; CVD: Cardiovascular disease; PCA: principal component analysis.

sTable 23. Sensitive analysis of the mediation of metabolic signature on the association of air pollution score and components with atrial fibrillation after using PCA to construct the air pollution score.

| Exposure | HR (95%CI) | *P* |
| --- | --- | --- |
| Air pollution |  |  |
| Direct effect | 1.11(1.08,1.13) | <0.001 |
| Indirect effect | 1.09(1.06,1.11) | <0.001 |
| Total effect | 1.02(1.02,1.02) | <0.001 |
| Mediation proportion (%) | 19.48(15.36,28.03) | <0.001 |
| PM_2.5_ |  |  |
| Direct effect | 1.08(1.06,1.13) | <0.001 |
| Indirect effect | 1.06(1.04,1.11) | <0.001 |
| Total effect | 1.02(1.02,1.02) | <0.001 |
| Mediation proportion (%) | 24.23(15.95,38.02) | <0.001 |
| PM_10_ |  |  |
| Direct effect | 1.12(1.09,1.14) | <0.001 |
| Indirect effect | 1.10(1.07,1.12) | <0.001 |
| Total effect | 1.02(1.02,1.02) | <0.001 |
| Mediation proportion (%) | 16.24(13.05,22.08) | <0.001 |
| NO_2_ |  |  |
| Direct effect | 1.11(1.08,1.12) | <0.001 |
| Indirect effect | 1.09(1.06,1.10) | <0.001 |
| Total effect | 1.02(1.02,1.02) | <0.001 |
| Mediation proportion (%) | 19.15(17.38,28.03) | <0.001 |
| NO_x_ |  |  |
| Direct effect | 1.11(1.09,1.14) | <0.001 |
| Indirect effect | 1.09(1.06,1.12) | <0.001 |
| Total effect | 1.02(1.02,1.02) | <0.001 |
| Mediation proportion (%) | 19.29(14.24,26.99) | <0.001 |

Models were adjusted for age, sex, race, BMI, physical activity, smoke, alcohol, DASH, history of diabetes mellitus, hypertension, CVD, and cancer. BMI: Body mass index; DASH: Dietary approaches to stop hypertension; CVD: Cardiovascular disease; PCA: principal component analysis.

**References:**

1. Beelen R, Hoek G, Vienneau D, Eeftens M, Dimakopoulou K, Pedeli X, et al. Development of NO2 and NOx land use regression models for estimating air pollution exposure in 36 study areas in Europe – The ESCAPE project. Atmos Environ[J]. 2013, 72:10-23.

2. Eeftens M, Beelen R, de Hoogh K, Bellander T, Cesaroni G, Cirach M, et al. Development of Land Use Regression models for PM(2.5), PM(2.5) absorbance, PM(10) and PM(coarse) in 20 European study areas; results of the ESCAPE project. Environ Sci Technol[J]. 2012, 46(20):11195-205.

3. Sun Q, Yang C, Jiang M, Yang M, Mao M, Fan Z. Ambient air pollution does not diminish the beneficial effects of active commuting on heart failure: a prospective study in UK Biobank. Bmc Public Health[J]. 2025, 25(1):828.

4. Yang L, Guo Y, Yao Y, Xie Y, Yang S, Shang B, et al. Circulating metabolomics revealed novel associations between multiple ambient air pollutants exposure and chronic obstructive pulmonary disease incidence: Evidence from a prospective cohort study. Environ Pollut[J]. 2024, 359:124727.
